# An integrated groundwater vulnerability and artificial recharge site suitability assessment using GIS multi-criteria decision making approach in Kayseri region, Turkey

**DOI:** 10.1007/s11356-024-33809-6

**Published:** 2024-06-04

**Authors:** Rachid Mohamed Mouhoumed, Ömer Ekmekcioğlu, Mehmet Özger

**Affiliations:** 1 Hydraulics Division, Civil Engineering Department, Istanbul Technical University, Istanbul, Turkey; 2 Energy and Environment Research Center, Faculty of Engineering, University of Djibouti, Balbala, Djibouti; 3 Disaster and Emergency Management Department, Disaster Management Institute, Istanbul Technical University, Istanbul, Turkey

**Keywords:** Managed aquifer recharge, DRASTIC, SWARA, FAHP, ROC curve, Sensitivity analysis

## Abstract

Groundwater resources worldwide face significant challenges that require urgent implementation of sustainable measures for effective long-term management. Managed aquifer recharge (MAR) is regarded as one of the most promising management technologies to address the degradation of groundwater resources. However, in urban aquifers, locating suitable areas that are least vulnerable to contamination for MAR implementation is complex and challenging. Hence, the present study proposes a framework encapsulating the combined assessment of groundwater vulnerability and MAR site suitability analysis to pinpoint the most featured areas for installing drywells in Kayseri, Turkey. To extrapolate the vulnerable zones, not only the original DRASTIC but also its multi-criteria decision-making (MCDA)–based modified variants were evaluated with regard to different hydrochemical parameters using the area under the receiver operating characteristic (ROC) curve (AUC). Besides, the fuzzy analytical hierarchy process (FAHP) rationale was adopted to signify the importance level of criteria and the robustness of the framework was highlighted with sensitivity analysis. In addition, the decision layers and the attained vulnerability layer were combined using the weighted overlay (WOA). The findings indicate that the DRASTIC-SWARA correlates well with the arsenic (AUC = 0.856) and chloride (AUC = 0.648) and was adopted as the vulnerability model. Groundwater quality parameters such as chloride and sodium adsorption ratio, as well as the vadose zone thickness, were found to be the most significant decision parameters with importance levels of 16.75%, 14.51%, and 15.73%, respectively. Overall, 28.24% of the study area was unsuitable for recharge activities with high to very high vulnerability, while the remaining part was further prioritized into low to high suitability classes for MAR application. The proposed framework offers valuable tool to decision-makers for the delineation of favorable MAR sites with minimized susceptibility to contamination.

## Introduction

The existing research recognizes that the global population is projected to experience a significant increase in the forthcoming years, with a predominant portion of individuals projected to inhabit urbanized regions (Lall et al. 2020). This substantial growth in population engenders significant challenges to fresh water resources, emphasizing the critical need for the adoption of sustainable water management practices to safeguard these vital resources (Bekele et al. 2018; Dillon et al. 2019). Despite elaborative management and acknowledgement of surface waters (e.g., rivers, streams, lakes), the sustainability of groundwater does not seem to have the same success (Howard 2015). Associatively, groundwater resources have been subject to severe challenges, including chemical contamination, depletion, anthropogenic activities, and seawater intrusion (Lall et al. 2020; La Vigna 2022) in recent decades. In addition to impacting the availability of subsurface resources, these challenges also have a detrimental effect on the quality of groundwater resources, rendering them less suitable for human consumption and irrigation.

An even more alarming situation exists in urban aquifers where groundwater extraction has grown at a rapid rate and natural recharge is limited by an increasingly drying climate combined with an explosive growth in urbanization (Dillon et al. 2019). The term “urban aquifer” refers to underground water reservoirs situated beneath urbanized regions. Although the science of urban groundwater is very young and still evolving, the pertinent literature covers significant scientific advances in various domains such as urban water balance, pollutant source characterization, contaminant migration assessment, managed aquifer recharge (MAR), monitoring well technologies, and mapping groundwater vulnerability (Howard 2015). Among the highlighted attempts, MAR represents a promising strategy to deal with most challenges (i.e., depletion, seawater intrusion, land subsidence, urban water management) faced by groundwater resources. The artificial recharge of groundwater or MAR involves injecting reclaimed water into the aquifer through different recharge structures for ensuring long-term recovery or environmental benefit (Bouwer 2002; IAH 2022).

The recharge method selected for a project depends not only on the site conditions and the infiltration performance of the selected MAR structure but also on its benefits and drawbacks. For instance, surface infiltration techniques, such as in-channel (dams placed across streams) and off-channel systems (i.e., infiltration basins), are recognized for their superior performance in contaminant attenuation among all MAR technologies. However, implementing these techniques necessitates the availability of permeable and spacious surface areas (Bouwer 2002). Due to pavements restricting permeability and urbanization that limits the land availability, surface infiltration techniques are less suitable in urbanized areas (Edwards et al. 2016). However, vadose zone recharge (VZR) techniques (especially drywells), which are small diameter shallow wells that end before reaching the water table, offer a promising solution for densely urbanized environments since they have negligible land impact, lead to minimal evapotranspiration losses, and can be installed in impermeable areas with limited financial resources (Händel et al. 2016; Edwards et al. 2016; Sasidharan et al. 2018).

Many scholars have highlighted the viability and efficiency of using small diameter shallow wells for conveying reclaimed water to the aquifer compared to infiltration basins which has been one of the mostly adopted artificial recharge mechanisms across the globe, recently. For instance, a comparison was performed by Händel et al. (2014) between the infiltration performances of drywells and surface basins using divergent numerical simulations under conditions that are commonly faced in field settings. The findings of the corresponding study indicated that a 10 m by 6 m infiltration basins and a group of two small-diameter vadose zone wells produced similar recharge rates. Likewise, Sasidharan et al. (2021) compared these two systems by further considering subsurface heterogeneity with the commercial HYDRUS (2D/3D) software and discovered that five drywells produce a higher infiltration rate than a 70-m diameter circular basin. Although drywells are well-regarded by the scientific community, the VZR mechanisms have been impeded by the potential risk of groundwater contamination due to the discharge of polluted stormwater runoff directly into the subsurface soil, circumventing the surface soil and near-surface sediment (Edwards et al. 2016). Despite being extensively adopted in the USA for several decades to alleviate stressed aquifers, there have been no reported instances of groundwater contamination linked to drywells (Brian 2018). Additionally, proper sitting and periodic maintenance of drywells were recommended by Edwards et al. (2016) in dealing with most contaminants found in urban stormwater, while contemporary drywell designs often incorporate pretreatment or sedimentation chambers (Lock et al. 2017; Edwards et al. 2022).

Nevertheless, the lack of guidelines and frameworks for identifying suitable sites for drywells hinders their global adoption. Despite numerous studies focusing on drywells, the pertinent literature has primarily concerned with their infiltration performances and clogging as well as the groundwater quality (Chahar et al. 2012; Liang et al. 2018; Glass et al. 2020; Justino et al. 2021; Edwards et al. 2022). Yet, none of these efforts neither devoted to assist in decision-makers with a comprehensive means of evaluating large areas for potential drywell construction nor allowing them to focus on regions that show promise with minimal financial and computational burden. In this vein, the identification of favorable sites for recharge structures is a critical step in the design of MAR project; however, it is especially worth mentioning that the numerous parameters involved in this process render it a highly challenging task (Rahman et al. 2012; Sallwey et al. 2019). Associatively, the pertinent literature have addressed the corresponding consideration with several methodologies, i.e., geophysical methods (Anbazhagan and Ramasamy 1997; Christy and Lakshmanan 2017), statistical analysis (Brown et al. 2016), and the integrated remote sensing and geographical information system (GIS) (Singh et al. 2013; Zaidi et al. 2015; Tiwari et al. 2017; Senthilkumar et al. 2019; Ahirwar et al. 2020).

Currently, the GIS-multi-criteria decision analysis (GIS-MCDA) is widely regarded as the most promising and emerging alternative for identifying suitable sites for the implementation of MAR techniques (Sallwey et al. 2019). The conflicts that arise from the presence of multi-disciplinary decision-makers, particularly when defining the relative importance of decision criteria in MAR projects, can render traditional Decision Support Systems (DSS) and GIS techniques ineffective (Rahman et al. 2012). Therefore, the integration of GIS and MCDA rationale, which warrants rewarding approaches to resolve multi-tiered decision-making processes (Ekmekcioğlu et al. 2021a), can yield more pragmatic outcomes that take both the spatial pattern of the decision alternatives and the value judgment of experts into account (Malczewski 2006). For instance, Sandoval and Tiburan (2019) used the analytical hierarchy process (AHP) technique along the GIS to delineate sites suitable for MAR structures to address the groundwater depletion in Mount Makiling Forest Reserve, Philippines. Aju et al. (2021) similarly adopted the AHP to identify possible sites to construct recharge facilities to tackle water scarcity in the Vamanapuram River Basin (VRB), South India. They found that rainwater infiltration pits and injection wells are suitable for the western and north-central zones of the basin, while percolation ponds and pond-cum injection wells were deemed appropriate for the central regions. Soliman et al. (2022) employed the AHP to identify feasible MAR sites for emergency scenarios for Nuweiba alluvial aquifer, Sinai, Egypt. Chowdhury and Paul (2021) coupled the fuzzy AHP (FAHP) and fuzzy gamma operator for determining the decision criteria importance level and integration of the thematic layers for the final MAR suitability map, respectively. Papadopoulos et al. (2022) proposed a hybrid FAHP based on the logarithmic fuzzy preference programming (LFFP) optimization method to prioritize and rank several sites based on their MAR suitability potential in an agricultural zone located in the southeast of Xanthi city, northern Greece. Based on their findings, the proposed methodology may be used to distinguish discrete preferable points (alternatives) within the MAR application without limiting the total number of alternatives analyzed. Moreover, recent attempts in the topic include the combination of multi-MCDA methods for the development of MAR suitability maps (Kamangar et al. 2019; Kharazi et al. 2019; Rajasekhar et al. 2021; Hussaini et al. 2021). Although the GIS-MCDA has extensively been adopted in order for examining the MAR technologies’ some variants, namely infiltration basins (Bonilla Valverde et al. 2016; Tsangaratos et al. 2017; Kazakis 2018; Zhang et al. 2019; Papadopoulos et al. 2022; Soliman et al. 2022), subsurface dams (Chezgi et al. 2016; Kharazi et al. 2019), rainwater harvesting (Singh et al. 2017; Chowdhury and Paul 2021; Tahvili et al. 2021), and injection wells (Ahani Amineh et al. 2017; Ghazavi et al. 2018; Sandoval and Tiburan 2019; Aju et al. 2021; Phankamolsil et al. 2022), however, to the best of the authors’ knowledge, there have been no attempts so far regarding their utilization for VZR wells suitability mapping, except the work of Mouhoumed et al. (2023).

Furthermore, while the GIS-MCDA assessment for identifying suitable sites for MAR implementation has demonstrated effectiveness, it is worth noting that the need to direct recharge efforts towards areas with the high effectiveness and low likelihood of vulnerability to contamination has still not been adequately addressed. Here, implementing the recharge structures in vulnerable zones may diminish the potential benefits and result in long-term negative impacts on groundwater quality. Hence, installation of the MAR structures in vulnerable zones may further necessitate water treatment, making the MAR project inefficient and costly. This is due to the possibility of recovered water requiring treatment prior to use for drinking or irrigation, with associated expenses and time requirements. Therefore, careful consideration of groundwater vulnerability is of paramount importance when selecting the optimal location for MAR structures in order to avoid the corresponding negative impacts and ensure the long-term viability and sustainability of the MAR project (Arshad et al. 2020).

In light of these considerations, the current research aimed to present a framework combining the assessment of MAR site suitability and the DRASTIC method (Aller et al. 1987), a widely utilized technique for mapping groundwater vulnerability (Patel et al. 2022). However, the conventional DRASTIC algorithm is prone to many criticism, arguing that modifying the features ratings and weights could enhance the model performances (Neshat et al. 2014a). Thus, several modified DRASTIC models were proposed in the literature discourse. To exemplify, many scholars coupled the DRASTIC method with MCDA algorithms, notably the AHP (Neshat et al. 2014a; Jhariya et al. 2019; Arshad et al. 2020; Bera et al. 2022), as well as statistical methods (Bonfanti et al. 2016; Ncibi et al. 2020; Siarkos et al. 2023). Moreover, artificial intelligence (AI) algorithms such as the artificial neural networks (ANN) (Bordbar et al. 2022; Smida et al. 2023), convolutional neural networks (CNN) (Dasgupta et al. 2024), support vector machines (SVM) (Ijlil et al. 2022), and random forests (RF) (Karimzadeh Motlagh et al. 2023) have been integrated with the original DRASTIC method to enhance model performance.

The particular objectives and the attempted contributions of this study to the body of knowledge can concisely be underpinned as follows:

- The identification of vulnerable areas within the aquifer using not only the original DRASTIC model but also its other versions modified by the MCDA-based approaches (i.e., AHP, FAHP, and SWARA).
- The comparison of the various vulnerability models by examining their relationships with the presence of different pollutants in the groundwater, and thereby selecting the outperforming model as the base vulnerability layer of the entire framework. Although numerous modified MCDA-based DRASTIC models have been proposed in the literature, their performances have rarely been comprehensively compared for the same study area. Therefore, this study undertakes the comparison of the performance of four distinct DRASTIC models. Furthermore, three distinct hydrochemical parameters were employed to evaluate their performance, representing a rare endeavor within the literature.
- The specification of most relevant decision variables to delineate the sites that are most favorable for drywell sitting and the determination of their individual significance level using FAHP in conjunction with a pairwise comparison survey involving experts from diverse backgrounds. To the authors’ best knowledge, the largest set of decision criteria spanning from surface, hydrometeorology, and groundwater protection clusters was used in this study to delineate sites suitable for drywells construction. Moreover, within the relevant literature, the fuzzified version of the AHP algorithm has been rarely applied (Mouhoumed et al. 2024).
- The adoption of sensitivity analysis to the FAHP in order to certify the stability and robustness of the decision framework. It involves the application of five distinct fuzziness degrees (i.e., 1.2, 1.4, 1.6, 1.8, and 2.0) in addition to the baseline fuzziness degree in the FAHP algorithm to assess potential shifts in the decision criteria rankings.
- The integration of the vulnerability and the decision layers to obtain the final suitability map that not only highlights unsuitable areas of the aquifer because of the vulnerability to groundwater contamination but also prioritizes the regions in terms of their MAR potential with respect to different degrees, i.e., low to medium. It is noteworthy that such a comprehensive integration, which accounts for both groundwater vulnerability and MAR site delineation, has not yet been proposed in the current literature discourse.

The outcomes of the present research provide insightful contributions to the research society and both decision and policy-makers regarding the deployment of robust and reliable MAR suitability maps that incorporate vulnerability assessment. Hence, it is believed that such maps can facilitate the sustainable management of aquifers and help address water scarcity challenges in different regions worldwide, while minimizing the risk of MAR failure caused by installing recharge facilities in areas prone to groundwater contamination.

## Description of the study area and problem statement

**Fig. 1:**
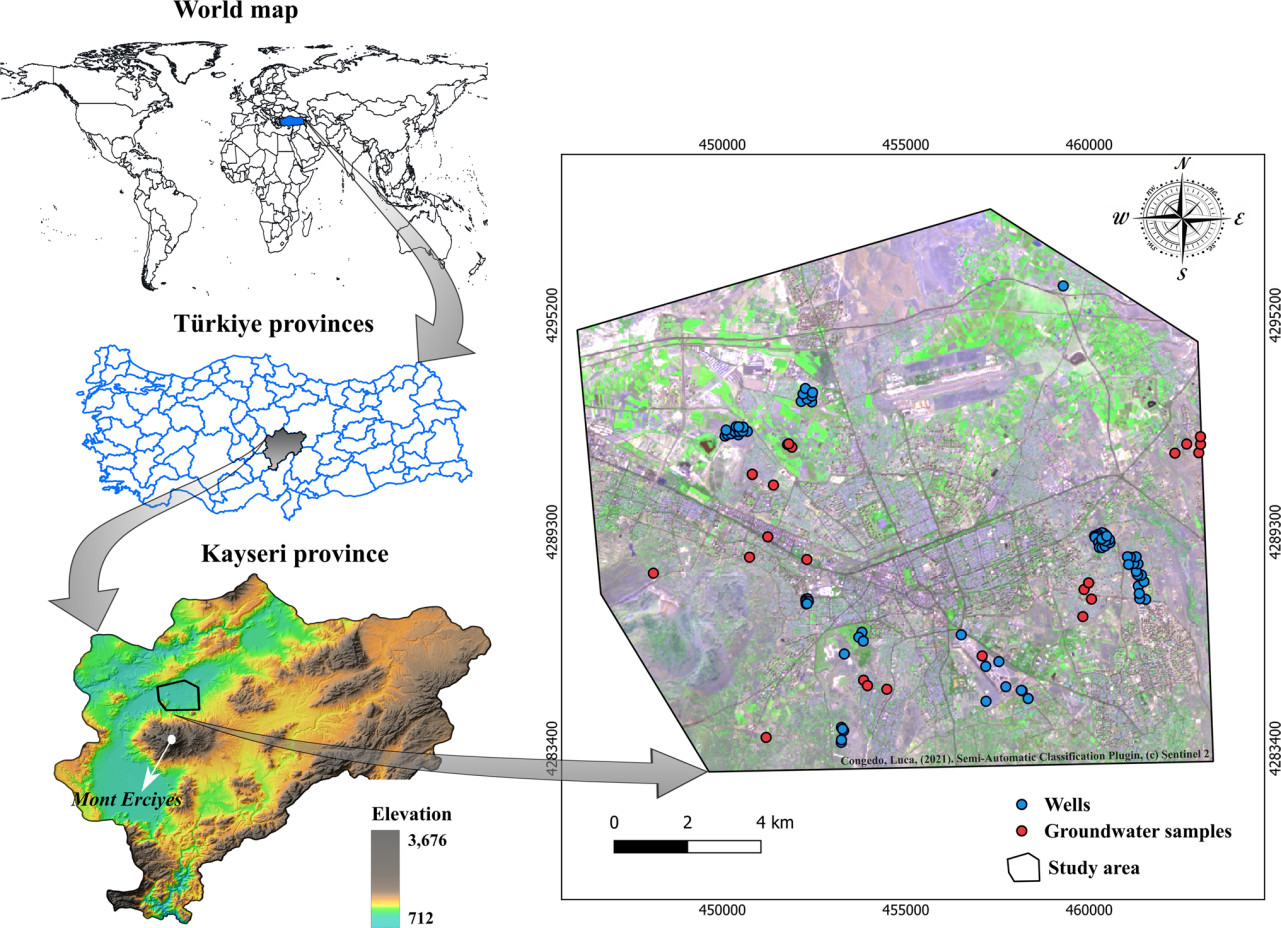
Study area (Kayseri city)

**Fig. 2:**
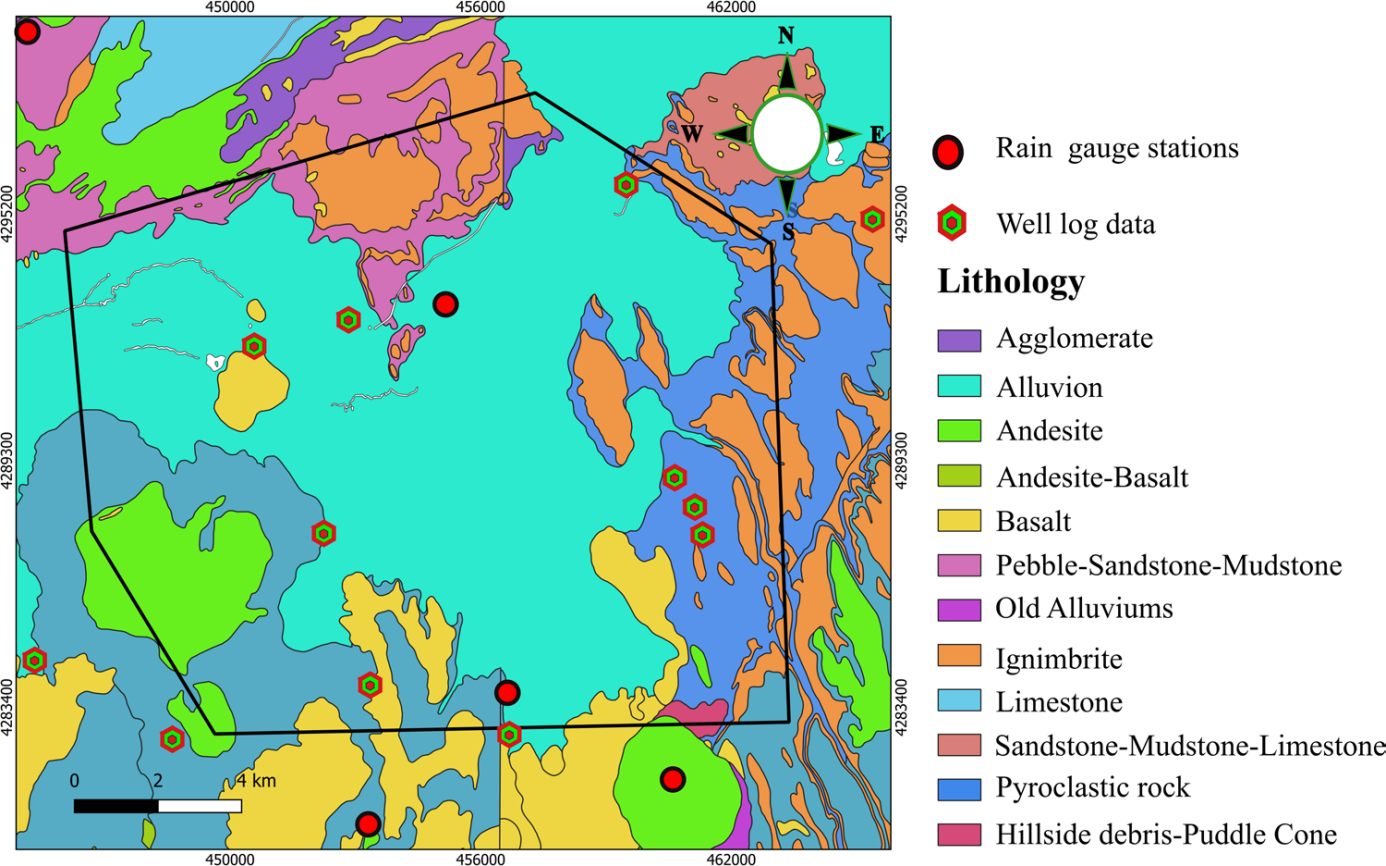
Lithology of the study area

This study concentrates on the metropolitan city of Kayseri, located in Central Anatolia, Turkey, with a population exceeding one million (Fig. 1). The study area covers a surface area of 218.53 km^2^ and includes Melikgazi and Kocasinan districts. The region is characterized by a cold semi-arid climate, with hot summers and mild winters. The average annual temperature is around 13 °C, with the warmest month being July with an average temperature of 25 °C and the coldest month being January with an average temperature of 2 °C. Precipitation is relatively low, with an average of around 400 mm per year (Turkish State Meteorology Service 2022). Geologically, Kayseri lies on the Central Anatolian Plateau, which includes various volcanic rocks that range in age from Paleocene to Paleo-Quaternary and reflect different hydrogeological characteristics (Elhatip et al. 2003). Additionally, the region is characterized by a number of active and inactive volcanoes, among which Mount Erciyes is the most prominent peak, towering at an elevation of 3916 m. Regarding the lithology, the study area is primarily composed of alluvial deposits, followed by pyroclastic rocks (Fig. 2). Additionally, other rock types such as andesite, basalt, ignimbrite, and sandstone are present within the region.

Beneath Kayseri city, two superimposed distinct aquifer systems are present, demarcated by an impermeable or semi-permeable layer predominantly comprised of andesite and basalt. The lower aquifer, characterized as a pressurized volcanic aquifer, primarily receives its recharge from snowmelt originating in the elevated regions of Erciyes mountain and from a deep spring with a flow rate of 300 l/s (Nuray Ates et al. 2021). Groundwater flows within this aquifer, as reported by Değirmenci et al. (2011a), predominantly occurs from the southern and southeastern directions towards the northern and northwestern directions, with a velocity range estimated at 15–20 cm/day. Positioned atop the confined aquifer is a shallow unconfined aquifer, which exhibits a significantly lower yield due to its thickness and extension (Yazıcı et al. 2015). This secondary alluvial aquifer is mainly feed by direct precipitation on the plain and the measured hydraulic conductivity varies between 1.1*10^−5^ and 6.4*10^−4^ m/s.

Moreover, the water requirements of Kayseri city heavily rely on its groundwater reservoirs. With the increasing population, industrialization, and agricultural activities, the demand for water in the city is rising, exacerbating the strain on already finite groundwater reserves and underscoring the necessity for sustainable water management strategies. It was reported that the municipality provided about 60 billion m^3^ of water for domestic use each year (Değirmenci et al. 2011b). It is noteworthy that most of this water is extracted from the lower pressurized aquifer. On the other hand, the upper aquifer is mainly used for agricultural purposes and has been suffering from a decline in water table due to lack of proper management activities. This depletion, coupled with the imperative to meet burgeoning agricultural water needs, raises concerns about potential over-extraction from the pressurized aquifer. As the lower aquifer is already vulnerable to climate change due to its reliance on the seasonal snowmelt discharge, excessive extraction could compromise both the quality and quantity of water. Therefore, it is imperative to adopt sustainable water management strategies to safeguard long-term water availability for urban and agricultural use, while addressing the risks of water scarcity and resource degradation.

An effective method for replenishing the shallow aquifer in the Kayseri region would be recharging it with stormwater runoff, which is an abundant source of water that could be reclaimed using shallow wells. Drywells are now one of the popular methods for stormwater management and aquifer recharge, and accordingly, numerous studies have explored their implementation recently (Sasidharan et al. 2018). Moreover, modern drywell such as the MaxWell Plus systems comes with dual pre-treatment chambers that pre-treats stormwater twice for constituent removal prior to infiltration (TorrentResources 2014), guaranteeing that the water being recharged into the aquifer is free of pollutants and other contaminants or at least with minimal concentration. Hence, in this research, the goal is to identify promising areas for constructing drywell structures in the city of Kayseri, using GIS-MCDA techniques.

## Materials and methods

The methodologies for conducting MAR suitability mapping in the literature are quite varied, as noted by Sallwey et al. (2019). However, recent studies have shown widespread acceptance of the procedure proposed by Rahman et al. (2012). This procedure consists of four key decision steps, which are as follows:

i. *Problem statement*
ii. : The initial step involves providing a general overview of the study area, clearly defining the purpose of the artificial recharge, and outlining the recharge techniques intended to address the identified problem (section “Description of the study area and problem statement”).
iii. *Constraint mapping*: The second step involves identifying and eliminating infeasible areas that may negatively impact the potential effectiveness of MAR before proceeding with the suitability assessment.
iv. *Suitability mapping*: The third step involves identifying relevant decision criteria, determining the significance level of each criterion, and integrating the thematic layers of various parameters to create a suitability map that prioritizes the study area.
v. *Sensitivity analysis*: The final step involves conducting a sensitivity analysis to evaluate and assess the robustness and reliability of the decision analysis framework.

**Fig. 3:**
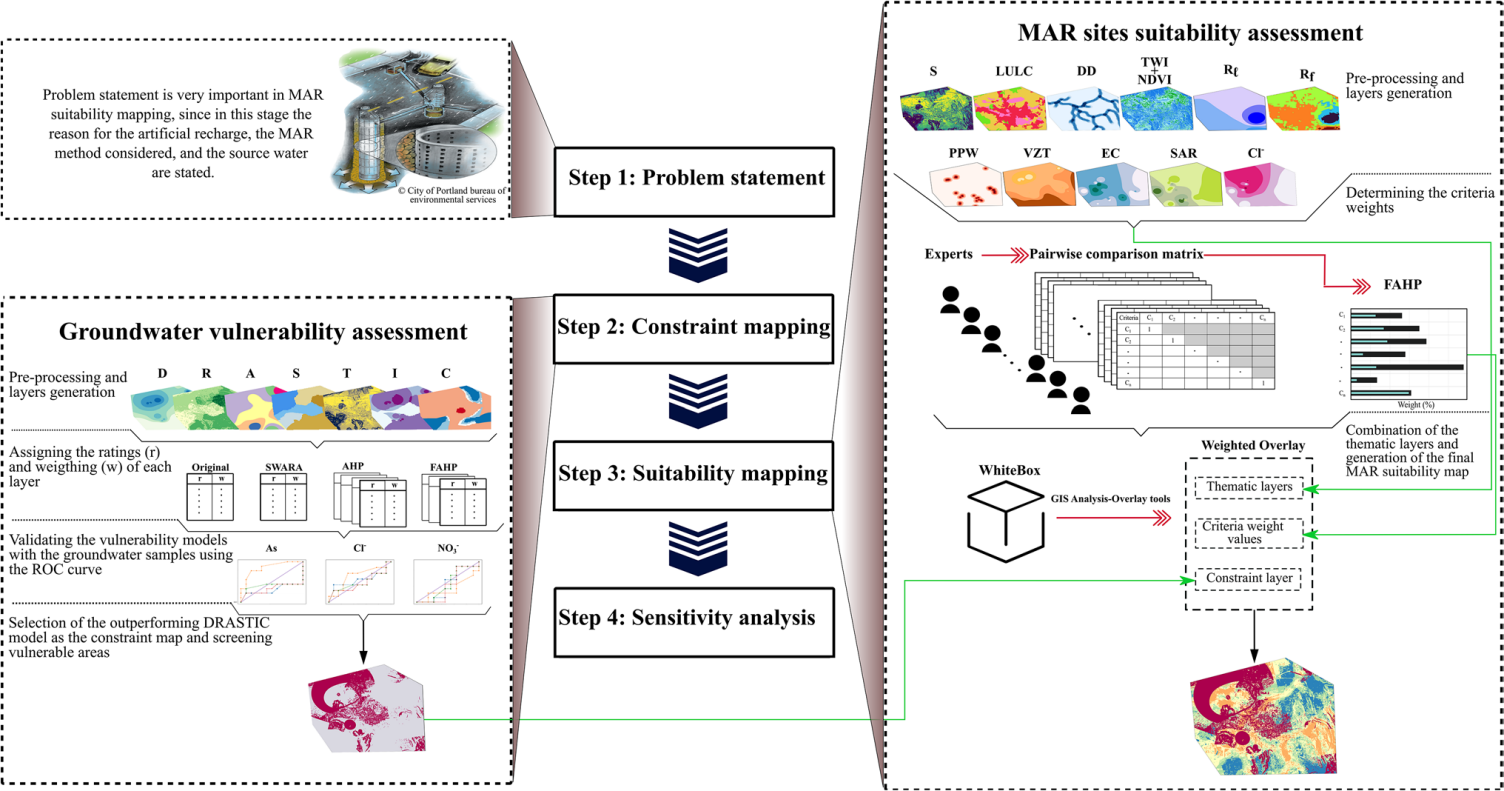
The framework proposed to integrate the DRASTIC model and the MAR site delineation assessment

In this study, we aimed to integrate the evaluation of groundwater vulnerability with the MAR sites suitability assessment, utilizing the procedure outlined above. The open-source QGIS software (QGIS Development Team 2022) was used for all spatial analysis in the present work. The framework utilized in this study, detailing the tasks assigned to each decision step, is presented in Fig. 3. The subsequent subsections provide a comprehensive account of each decision step, elaborating on the methodology embraced in this paper for the purpose of offering a clear depiction of the approaches conducted in this research.

### Site screening

**Fig. 4:**
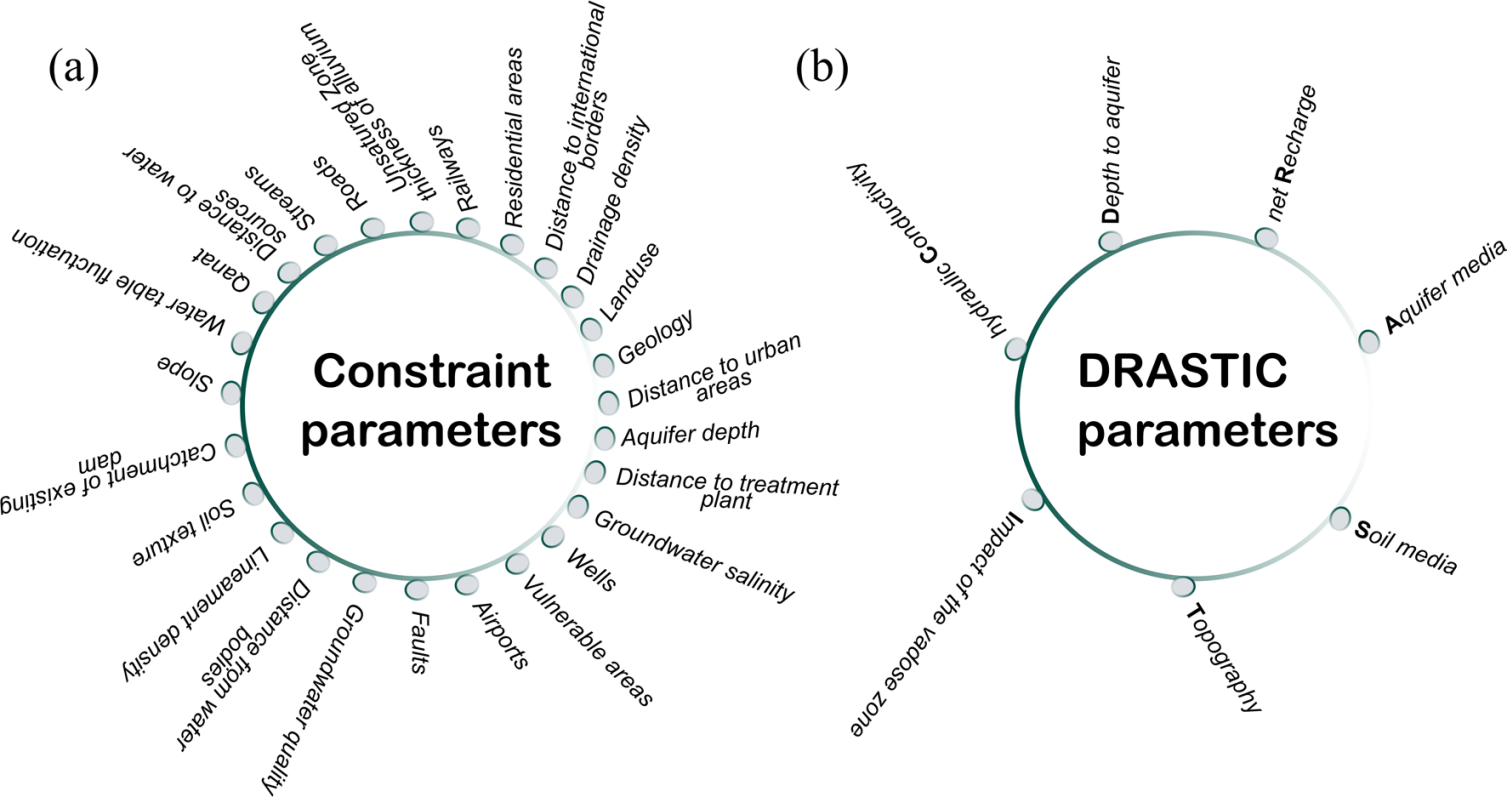
List of **a** constraint parameters adopted so far in MAR site suitability mapping studies and **b** the DRASTIC model factors

The constraint mapping phase is an important step in identifying suitable areas for aquifer recharge, during which areas that are not suitable for recharge are eliminated from further consideration. Sallwey et al. (2019) conducted a comprehensive review analysis encompassing 63 studies employing GIS-MCDA methods for MAR suitability mapping, revealing that 48% of these studies utilized constraint mapping as a pivotal tool for screening out unfeasible areas. In Fig. 4a, we presented all the criteria used in the literature to perform the site screening process. One can observe that most of these criteria (such as slope, land use characteristics) are straightforward to generate. However, attaining information regarding the potential vulnerability of an area can be challenging in the absence of groundwater vulnerability model utilizations. Therefore, the DRASTIC method was incorporated into this research as a constraint mapping tool to identify and eliminate highly vulnerable areas before acknowledging the suitability mapping attempts. The DRASTIC method is a widely recognized technique that combines and evaluates the intrinsic vulnerability of an aquifer through the examination of its physical and hydrogeological characteristics using an overlay and index approach (Aller et al. 1987). The corresponding model has extensively been used over the past years, globally, as a groundwater pollution assessment tool (Khosravi et al. 2018; Hasan et al. 2019; Soyaslan 2020; de León-Gómez et al. 2021). The acronym “DRASTIC” stands for the seven hydrogeological factors, called depth to water (*D*), net recharge (*R*), aquifer media (*A*), soil media (*S*), topography (*T*), impact of the vadose zone (*I*), and hydraulic conductivity (*C*) (Fig. 4b). In order to determine vulnerability mapping, the DRASTIC index (DI) is calculated as the weighted sum overlay of the factors listed above, as shown in the equation below:

1 $$DI={D}_{r}{D}_{w}+{R}_{r}{R}_{w}+{A}_{r}{A}_{w}+{S}_{r}{S}_{w}+{T}_{r}{T}_{w}+{I}_{r}{I}_{w}+{C}_{r}{C}_{w}$$

where “$r$” and “$w$” denote the ratings and weights, respectively, that are assigned to each individual factor.

#### Preparation of the vulnerability model parameters

**Fig. 5:**
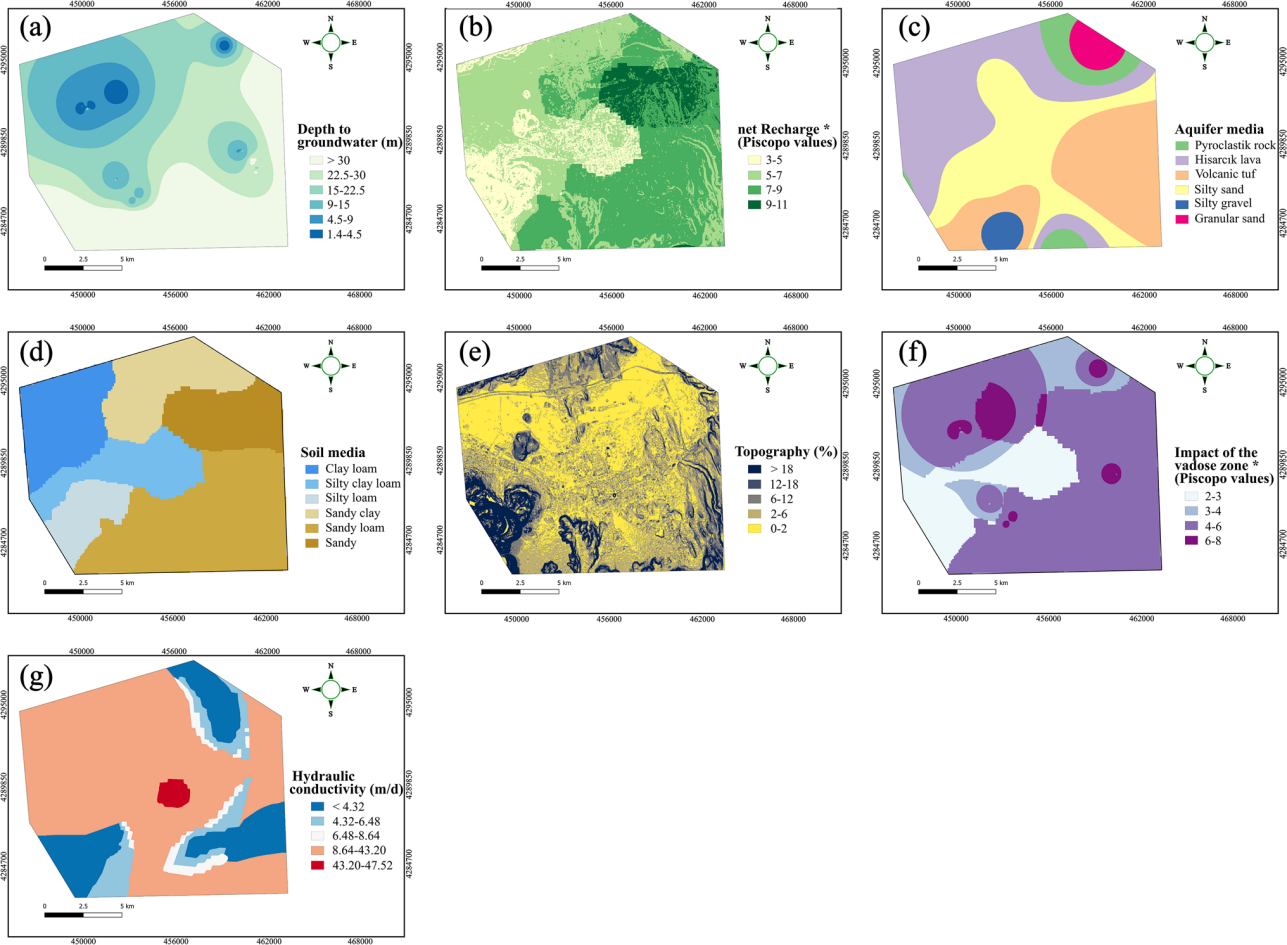
Thematic layers of the DRASTIC model. **a** Depth to water table, **b** net recharge, **c** aquifer media, **d** soil media, **e** topography, **f** impact of the vadose zone, and **g** hydraulic conductivity

The depth to water table is a crucial factor in evaluating intrinsic vulnerability as it relates to accessibility and potential contamination of groundwater. It also influences the duration of contact between contaminants and surrounding materials, as well as the potential for attenuation (Aller et al. 1987). Greater depth to water table provides longer travel times for contaminants making them undergo attenuation. The corresponding layer was obtained from the water table measurements from wells in the Kayseri plain and interpolated using the inverse distance weighting (IDW) as shown in Fig. 5a.

Net recharge refers to the quantity of rainwater that infiltrates into the ground and reaches the groundwater table (Patel et al. 2022). Recharge water, therefore, facilitates the transport of solid or liquid contaminants to the water table. Accordingly, regions with higher precipitation rates are more vulnerable to potential contamination potential compared those characterizing lower precipitation regimes. The net recharge of the study area was obtained through the Piscopo (2001) method (Fig. 5b). The following equation is used to derive the net recharge layer:

2 $$\text{Net recharge}=\text{Slope }\left({\%}\right)+\text{Rainfall}+\text{Soil permeability}$$

where slope was obtained from the Copernicus global digital surface model (Copernicus DSM GLO), provided by the European Space Agency and Sinergise (2021). The average rainfall was estimated and interpolated from the recording rain gauge stations located within the region. The soil permeability was obtained from the soil texture map used by Değirmenci et al. (2011a) in their research. Furthermore, Table 1 exhibits the scores and ratings assigned to each parameter based on the Piscopo (2001) approach.

**Table 1:** Net recharge and impact of the vadose zone assigned ratings based on Piscopo approach (Piscopo 2001)

Parameters	Range	Rating
Slope^a^ (%)	< 2	4
	2–10	3
	10–33	2
	> 33	1
Rainfall^a^ (mm)	> 850	4
	700–850	3
	500–700	2
	< 500	1
Soil permeability^a,b^	High	5
	Mod–high	4
	Moderate	3
	Low	2
	Very low	1
Depth to water table^b^ (m)	< 5	5
	5–10	4
	10–15	3
	15–20	2
	> 20	1
Net recharge	11–13	10
	9–11	8
	7–9	5
	5–7	3
	3–5	1
Impact of the vadose zone	8–10	10
	6–8	8
	4–6	5
	3–4	3
	2–3	1

^a^Parameters used to generate the net recharge layer

^b^Parameters used to generate the impact of the vadose zone layer

The aquifer medium, which refers to the geologic material that comprises an aquifer, plays a crucial role in determining the flow system and path length of groundwater within the aquifer (Aller et al. 1987). These factors are important in assessing the time available for various attenuation processes to occur, such as sorption, reactivity, and dispersion (Piscopo 2001). In the present study, the aquifer media layer was generated from 12 well logs data provided by the general directorate of water and sewerage administration of Kayseri municipality. The corresponding map is presented in Fig. 5c.

The soil media denoting the type of soil that overlies the aquifer also regarded as another important component of the DRASTIC approach as it is used in determining the rate at which contaminants migrate into the aquifer from the soil. Different soil types have varying infiltration rates that have significant impact on the transport of contaminants into the aquifer. For instance, soil with a high clay content may exhibit lower permeability, which could impede the migration of contaminants in comparison to soils encompassing coarse-grained textures, such as sand or gravel (Umar et al. 2022). In this study, a soil texture map of the study area was obtained from Değirmenci et al. (2011a) and digitized to further aid in the assessment of groundwater vulnerability (Fig. 5d).

The topography of an area influences the slope of the land surface and thereby affects the fate of contaminants by either facilitating rapid runoff or prolonged period, enabling the infiltration (Aller et al. 1987). Therefore, areas with lower slope tend to have a greater potential for pollutants to percolate through the soil and reach the water table compared to steeper regions. The slope of the study area was determined from the Copernicus DSM GLO with 30-m resolution using the OpenTopography DEM downloader pluging (European Space Agency and Sinergise 2021) in QGIS and the corresponding map is presented in Fig. 5e.

The vadose zone, also known as the saturated zone, refers to the subsurface above the water table. The attenuation potential of the vadose zone is highly influenced by the soil characteristics; such that factors such as the grain size of soil and the depth of the water table have a significant impact on the protective capacity of this zone. Areas with coarse soil and shallow water tables tend to be more vulnerable to pollutants, while those with finer soil and deeper water tables have lower vulnerability (Hussain et al. 2017). In this paper, the impact of the vadose zone layer was also determined through the Piscopo (2001) approach by using the following equation:

3 $$\text{Impact of the vadose zone}=\text{Soil permeability}+DTWT$$

where $DTWT$ refers to the depth to water table. In Table 1, the scores and ratings assigned to the corresponding factors are further presented.

The hydraulic conductivity is a fundamental parameter characterizing the ability of the subsurface materials to transmit water, and it plays a pivotal role in determining the flow dynamics of contaminants within the aquifer (Karan et al. 2018). Areas with higher hydraulic conductivity are more susceptible to increased transportation of pollutants, resulting in escalated vulnerability to groundwater contamination. The hydraulic conductivity layer was obtained from the study conducted by Değirmenci et al. (2011a) and digitized as shown in Fig. 5g.

#### Ratings and weighting

In this study, in addition to the original DRASTIC model proposed by Aller et al. (1987), various modified vulnerability assessment models proposed in the relevant literature were also taken into consideration in order to manifest a comprehensive understanding. Several theories on the origin of DRASTIC approach have been proposed to improve its performance by modifying its ratings and weights, leading to a more precise vulnerability assessment (Neshat et al. 2014b; Patel et al. 2022). Therefore, the weights and ratings adopted in three modified MCDA-based DRASTIC models, namely the analytic hierarchy process (AHP), fuzzy AHP, and the stepwise weight assessment ratio analysis (SWARA), were retrieved from the existing literature in order to obtain a rigorous vulnerability map. The ratings and weights assigned in those studies were grouped, averaged, and normalized, and the outcomes of this process are presented in Table 5.

The standard AHP, developed by Saaty (1988), is one of the most popular MCDA methods in many field of science. The AHP decomposes complex decision-making problems into hierarchical structures. For instance, in the case of the DRASTIC method, the objective of the model which is in fact the determination of the DRASTIC index will be at the first level of the hierarchy, while the DRASTIC layers will be located at the second level of the hierarchy. Relative importance values to each criterion in the hierarchy are determined as a result of the pairwise comparisons. This process enables the calculation of each decision parameter weight, thereby providing a consistent and transparent basis for decision-making. The fuzzy analytic hierarchy process (FAHP), as an extension of the standard AHP, addresses some of the limitations of the original non-fuzzy method (Laarhoven and Pedrycz 1983). Instead of using a crisp scale, the pairwise comparison survey in the FAHP utilizes linguistic variables, which are transformed into fuzzy numbers using different membership functions, including triangular, trapezoidal, and Gaussian functions. This incorporation of fuzzy set theory into the AHP presents a unique opportunity to address the inherent uncertainty and subjectivity associated with human decision-making processes. This approach, by offering nuanced and flexible features, takes the imprecision and vagueness encountered in decision processes into account, allowing more reliable decision outcomes. A more comprehensive explanation of the functionality and mathematical foundations of the FAHP can be found in the suitability mapping subsection (Section 3.2.2).

The SWARA method that was initially introduced by Keršulienė et al. (2010) is regarded as an expert-oriented method encompassing a ratio analysis approach compared to a more structured rationale embraced in the AHP technique. A key peculiarity of the SWARA comes from the role of decision makers since each expert is allowed to choose the importance of each criterion and ranks all the criteria from the first to the last one based on their implicit knowledge, information, and experiences (Zolfani and Saparauskas 2020). Additionally, the SWARA method offers a notable advantage over other MCDA techniques (such as AHP) since the SWARA enables assessing a large number of criteria with fewer pairwise comparisons required, whereas the AHP could necessitate a substantial number of pairwise comparisons in such multi-dimensional problems (Stanujkic et al. 2015). This feature of the SWARA makes it particularly advantageous in cases where the number of criteria is substantial, reducing the burden of the decision-making process. An in-depth understanding of the SWARA process can be attained from Torkashvand et al. (2021).

#### Comparison and validation of the vulnerability maps

To access and compare the accuracy of the traditional and modified DRASTIC models, the receiver operating characteristic (ROC) curve was used and the area under the curve (AUC) was computed for a comprehensive performance evaluation. Although nitrate is one of the most commonly used pollutants for validating the DRASTIC model (Khosravi et al. 2018; Jhariya et al. 2019; Kumar and Pramod Krishna 2020; Mallik et al. 2021), multiple chemical contaminants were also considered in this study to identify a clear picture regarding the outperforming vulnerability model. Hence, the concentrations of the nitrate (NO_3_^−^), arsenic (As), and chloride (Cl^−^) were collected from 23 groundwater samples that exist within the boundaries of the focalized region. The vulnerability indices generated by the DRASTIC models were retrieved at the sample locations using the point sampling tool available in QGIS. These indices, combined with the hydrochemistry data (NO_3_^−^, As, and Cl^−^), were utilized to produce different ROC curves.

The AUC ensures the overall comparison of the adopted approaches. Regarding the corresponding indicator, a perfect model accounts for a value of 1, pinpointing that the utilized approach produces a true positive rate of 1 and a false positive rate of 0 concerning all instances. On the other hand, a random model can take at least 0.5 reflecting that it has no ability to identify the distinctions between the classes. Being one of the widely accepted and informative metrics to yield the discriminatory nature of the adopted techniques, it provides a single scalar value that summarizes the overall performance of the model adopted, making it a feasible metric to ensure fair comparison datum line (Chen et al. 2020).

The *Y*-axis of the ROC curve represents the true positive rate (sensitivity), which is the rate of correctly classified instances, while the *X*-axis represents the false positive rate (1-specificity), which denotes the rate of incorrectly classified instances (Torkashvand et al. 2021). In this vein, the ROC curve allows for the identification of the trade-off between sensitivity and 1-specificity across different threshold values. A model with a ROC curve closer to the top left corner yields a higher AUC value indicating a greater ability of the model to distinguish within the conflicting instances. Furthermore, the AUROC can be computed as follows:

4 $$AUC=1-\frac{1}{{m}^{+}{m}^{-}}\sum_{{x}^{+}\epsilon {M}^{+}} \sum_{{x}^{-}\epsilon {M}^{-}}\left(\left(f\left({x}^{+}\right)<f\left({x}^{-}\right)\right)+\frac{1}{2}\left(f\left({x}^{+}\right)=f\left({x}^{-}\right)\right)\right)$$

where ${m}^{+}$ and ${m}^{-}$ are positive and negative instances, ${M}^{+}$ and ${M}^{-}$ are set of all positive and negative instances, and ${x}^{+}$ and ${x}^{-}$ are positive and negative classes, respectively. In the equation, $f\left(x\right)$ denotes the outcome of utilized approach with respect to instance $x$.

### Suitability mapping

The suitability mapping phase constitutes a pivotal aspect of the assessment of potential MAR sites. This process involves the collection of relevant decision factors that may impact the suitability of an area for the MAR implementation, and the allocation of weights to each criterion based on its relative significance in determining appropriate locations for the MAR construction. This study identified 11 decision criteria (i.e., slope, land use/land cover, drainage density, potential occurrence of shallow groundwater, rainfall, runoff, proximity to public wells, vadose zone thickness, electrical conductivity, sodium absorption ratio, and chloride) through an extensive literature review encompassing research articles and technical reports regarding drywells, which is the MAR technology considered. The refined list of criteria was grouped into four main clusters: *surface*, *hydrometeorology*, *groundwater protection*, and *quality* in order for constructing a better hierarchical representation. Subsequently, a pairwise comparison survey was conducted involving experts from diverse backgrounds, and the FAHP method was employed to determine the criteria weights. The remaining parts of this subsection provide a detailed overview of each layer employed for the suitability assessment (Section 3.2.1), as well as the architecture of the FAHP method (Section 3.2.2).

#### Preparation of the MAR site suitability decision layers

Sallwey et al. (2019) addressed that the slope is one of the most used criterion in MAR sites suitability mapping. The slope of the land surface plays a crucial role in the convergence and divergence of water, affiliating with the infiltration capacity of the soil (Ahmadi et al. 2017). Furthermore, clogging in drywells caused by suspended sediments in stormwater is a significant issue that arises from soil erosion in regions with steep slopes. In this research, the map produced for the soil layer was derived from the Copernicus DSM GLO dataset and further preprocessed using QGIS as presented in Fig. 7a.

Land use/land cover (LULC) is another critical parameter in the assessment of sites favorable for VZR structures. LULC is of paramount importance in the accurate prediction of different stormwater pollutant types that pose divergence with regard to different land use types surrounding drywell sites and having drastic impacts on the aquifers. To accurately predict the presence of specific stormwater contaminants and their impact on the local groundwater system, it is crucial to gather comprehensive information about the land use type in the vicinity of the drywell site (Edwards et al. 2016). The CORINE land cover (CLC) dataset (Büttner et al. 2017) was used to generate the LULC layer and the corresponding map is presented in Fig. 7b.

Drainage density (DD) is described as the proportion between the total length of all stream segments within the specified region of interest and the surface area (Arshad et al. 2022). This factor exhibits an inverse relationship with permeability, meaning the lower DD in a region the higher the permeability, resulting in increased infiltration and reduced surface runoff. Conversely, regions with high DD are more susceptible to not only erosions but also low infiltration rates and significant surface runoff. The DD map of the study area is displayed in Fig. 7c.

5 $$DD=\frac{\Sigma L}{A}$$

where ∑*L* represents the cumulative length of all stream segments within the area of interest, and *A* signifies the total surface area of the drainage basin.

**Fig. 6:**
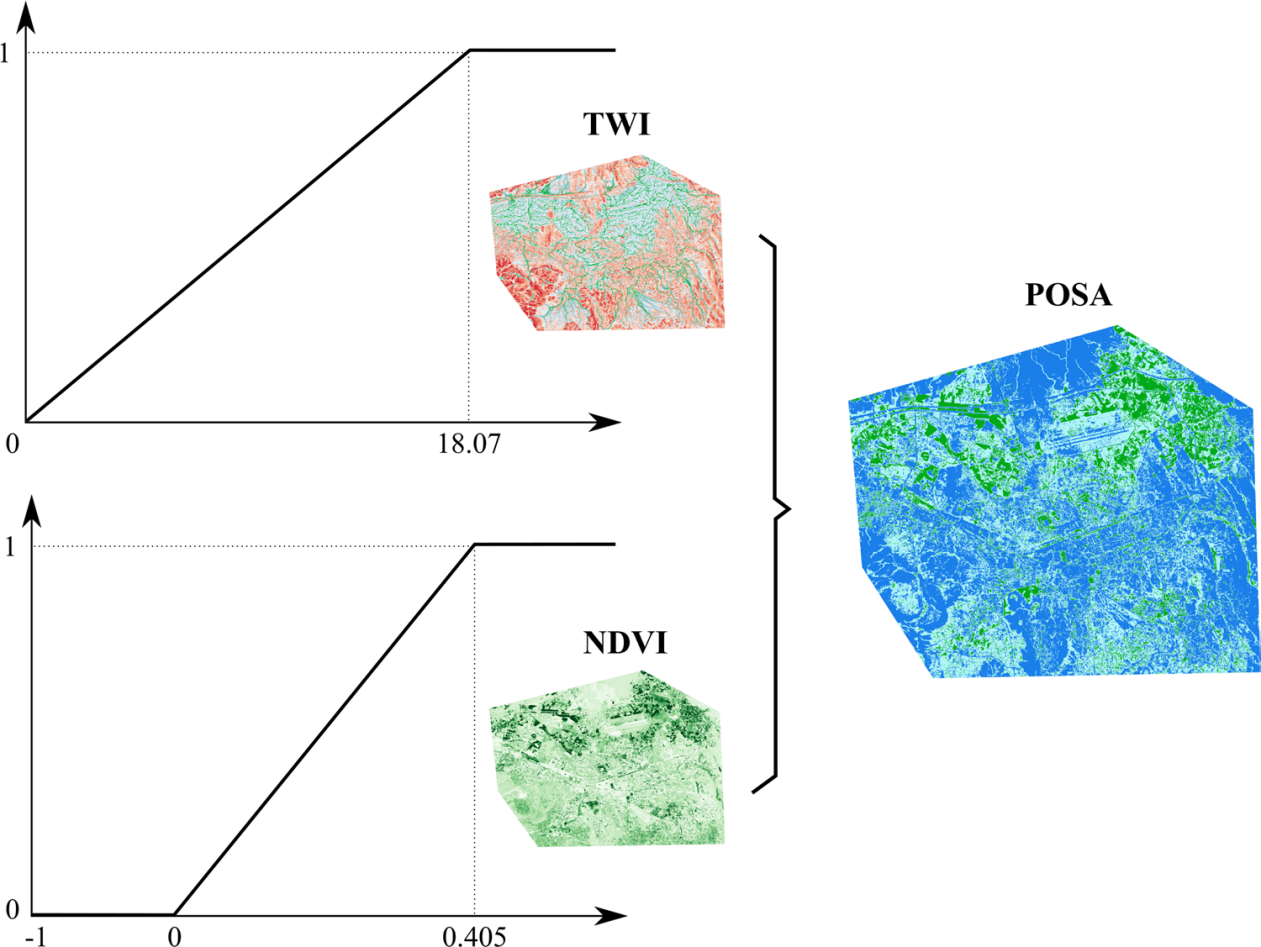
Linear fuzzy membership functions adopted for the TWI and NDVI layers to obtain the POSA layer

**Fig. 7:**
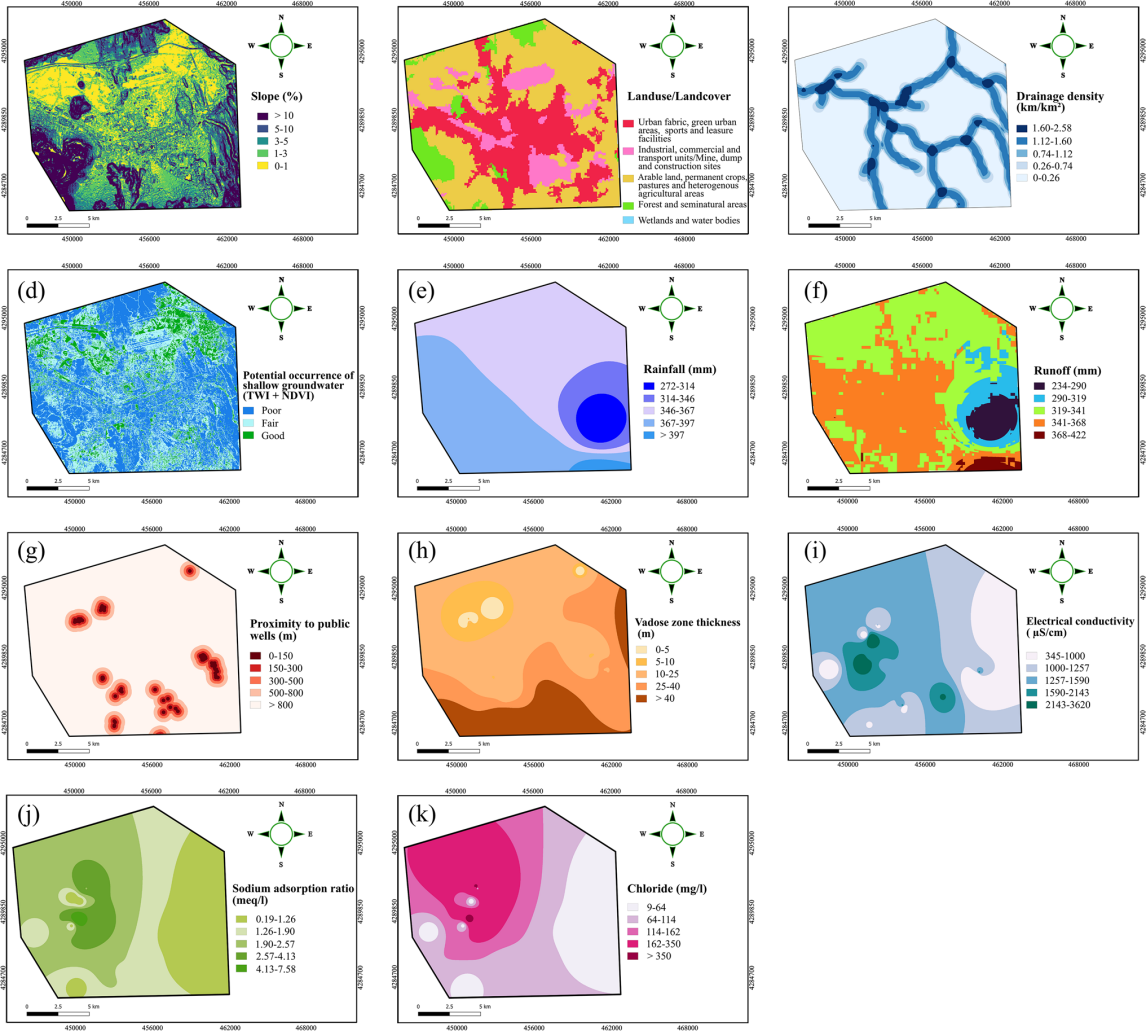
Thematic layers generated for the suitability mapping assessment. **a** Slope, **b** land use/land cover, **c** drainage density, **d** POSA, **e** rainfall, **f** runoff, **g** proximity to public wells, **h** vadose zone thickness, **i** electrical conductivity, **j** sodium adsorption ratio, and **k** chloride

The extend of the shallow aquifer in the Kayseri plain remains uncertain due to limited investigations and sparse documentation, highlighting the need for further research to determine its distribution and characteristics. Hence, to ensure the success of the recharge process, it is of utmost significance to implement drywells within the boundaries of the alluvial aquifer. To address this, a layer called the potential occurrence of shallow aquifer (POSA) was generated by combining the topography wetness index (TWI) and the normalized difference vegetation index (NDVI). Areas with high TWI and high NDVI could be used as an indicative of the presence of shallow groundwater, as such areas exhibit favorable topographic attributes conducive to water accumulation, coupled with limited vegetation cover facilitating enhanced infiltration processes (Ansems et al. 2015). Conversely, areas with low TWI values and high NDVI values may suggest regions where groundwater recharge potential is lower due to steeper slopes and denser vegetation cover, limiting infiltration. It is worth mentioning that this research adopted the fuzzy linear membership functions to standardize the TWI and NDVI layers (as presented in Fig. 6), and the sum overlay tool was used to generate the POSA layer (Fig. 7d).

Rainfall (*R*_l_) is another crucial parameter in the assessment of sites suitable for MAR technologies, as the variations in rainfall intensity regulate the hydrologic processes such as runoff, stream flow, and overland flow (Kaliraj et al. 2015). It is, therefore, the rainfall associates with the drywell projects, especially where the primary source of water is the stormwater runoff. Thus, average annual rainfall series were obtained from different meteorological measurement stations in not only Kayseri but also the regions located in its vicinity. Based on the acquired rainfall values, the finalized rainfall map was produced (Fig. 7e) by means of the inverse distance weighted (IDW) as it is one of the commonly applied and accurate interpolation technique (Gong et al. 2014).

In addition to their usefulness for recharging stressed aquifers, drywells serve as effective low impact development (LID) strategies to mitigate surface runoff. Areas experiencing higher levels of runoff are prioritized for the installation of VZR structures (Ghazavi et al. 2018). In this research, the runoff map was determined using the curve number method, pioneered by US Soil Conservation Service (SCS), through the following equation:

6 $$Q=\frac{{(P-0.2S)}^{2}}{(P+0.8S)}$$

with,

7 $$S=254\left(\frac{100}{CN}-1\right)$$

where $P$ and $CN$ are the precipitation and the curve number, respectively. The $CN$ of the study area was obtained from the global curve number (GCN250) datasets with 250-m resolution (Jaafar and Ahmad 2019).

The guidelines regarding the installation of drywells highlight that the drywell designs necessitates locating at a sufficient distance from production wells to permit adequate travel time to the recharged water (Geosyntec consultants 2020). In the USA, where the use of drywells is prevalent, there is a legally mandated separation distance between drywells and public supply wells. However, it is noteworthy that this threshold distance may vary based on the mandates of each state (Lock et al. 2017). For instance, a minimum horizontal distance of 60 m from drinking wells is required in Illinois, while a distance of at least 150 m is mandatory in Oregon (Geosyntec Consultants 2018). In this research, a proximity to public wells (PPW) map (Fig. 7g) was derived by taking the point coordinates of production wells using the proximity (raster distance) tool in QGIS.

Likewise, guidelines governing the installation of drywells mandate a minimum separation distance between the drywells and the seasonal high water table (County of Los Angeles 2009; Cities and County of Riverside 2012; Lock et al. 2017; ADEQ 2018), owing to the potential advantages conferred by a thick vadose zone in mitigating pollutants. The thickness of the vadose zone acts as a natural filter that retains pollutants within the zone and inhibits their migration into the underlying aquifer. Figure 7h illustrates the map of the vadose zone thickness.

The injection of reclaimed water in an area with compromised groundwater quality could jeopardize the recharged water, and thereby undermine the benefits of recharging or necessitate additional treatment prior to reuse (Ahani Amineh et al. 2017). Thus, assessing the quality of pre-existing water in the aquifer is imperative for successful MAR implementations. To address this issue, the current study aimed to evaluate the groundwater quality in the Kayseri alluvial aquifer by using three key parameters, i.e., electrical conductivity (EC), chloride (Cl^−^), and sodium adsorption ratio (SAR). Here, EC indicates the salinity and mineral content of water in terms of the ability to conduct electrical current. Insight on the water’s origin and contamination level in groundwater can be determined through Cl^−^ concentration. The SAR parameter is used to evaluate the ratio of sodium concentration (Na^+^) in one specimen to the total concentration of calcium (Ca^+^) and magnesium (Mg^2+^) and is a crucial factor in determining the suitability of the water for irrigation activities (Sattari and Feizi 2020). The following formula is used to calculate SAR:

8 $$SAR=\frac{{Na}^{+}}{\sqrt{\frac{1}{2}\left({Ca}^{2+}+{Mg}^{2+}\right)}}$$

where the concentrations of Na^+^, Ca^+^, and Mg^2+^ are expressed in milliequivalents per liter. The corresponding hydrochemical parameters were obtained from the study conducted by Değirmenci et al. (2011a) and the IDW interpolation technique was performed to generated their maps as shown in Fig. 7i, j, and k, respectively.

#### Fuzzy analytical hierarchy process

The analytical hierarchy process (AHP), first introduced by Saaty (1977), is a widely used method for hierarchical representation of complex problems based on the subjective opinions of experts. The AHP allows for both qualitative and quantitative evaluations, including criteria weighting and prioritizing alternatives. Despite its strengths, such as its user-friendly structure and robustness from various applications (Ekmekcioğlu et al. 2021b), the traditional AHP can be limited by its lack of consideration for uncertainty and imprecision in human judgment (Koc et al. 2021). The FAHP, on the other hand, addresses these limitations by incorporating the fuzzy set theory, pioneered by Zadeh (Zadeh 1965), to handle the complex and imprecise nature of the environment and experts’ preferences. The fuzzy AHP uses linguistic variables to represent experts’ preferences, allowing for the representation of uncertainty and imprecision in the decision-making process. The FAHP replaces the pairwise comparison matrices of the traditional AHP with fuzzy numbers, which are fuzzy subsets of real numbers. The weight determination process is carried out by determining the extent of each fuzzy number’s membership in a given fuzzy set. The FAHP steps facilitated in this study can be expressed as follows:

1. **Collection of data:** In order to establish a hierarchical structure in the study, 10 participants (Table 3) were asked to evaluate decision criteria in pairs using linguistic variables on a 1–9 comparison scale. Among various membership functions (e.g., triangular, gaussian, trapezoidal, sigmoid) to incorporate into the criteria weight computations (Sahin and Yip 2017; Lyu et al. 2020), the current research employed the triangular fuzzy numbers due to its several benefits, such as simple and intuitive structure, handling ambiguity and subjectivity, and common adoption (Ekmekcioğlu et al. 2022a). The reciprocals of linguistic variables regarding importance degrees of the criteria were incorporated into the preferences of the experts attended the surveys. Thus, ${l}_{ij}$, ${m}_{ij}$, and ${u}_{ij}$ indicating the lower, mean, and upper width of the pairwise judgments of the experts for criterion $i$ compared to the criterion $j$, respectively, are determined (Table 2).
2. **Checking consistency of the experts:** Ensuring the reliability of expert preferences is one of the key aspects of the AHP technique. The consistency checks are achieved by calculating the consistency ratio (CR), in which CR values greater than 0.1 indicate inconsistent judgments by respondents, while lower CRs than the corresponding threshold imply more consistent set of expert preferences (Table 3). Following expression can be used in identifying the CR values:
  9 $$CR=\frac{\frac{{\lambda }_{\text{max}}-n}{n-1}}{RI}$$
  where ${\lambda }_{\text{max}}$ and $n$ denote the maximum eigenvalue of the matrix and the number of criteria in the matrix, respectively. In addition, $RI$ is the random index, which was introduced by Saaty (2004), grounding on the size of the matrix (Table 4).
3. **Aggregating the group decisions:** The determination of fuzzy equivalents of linguistic expressions is accounted for in this step. Let the individual expert represented by $k$ and the study examines the triangular fuzzy membership, fuzzy equivalents, i.e., lower, mean, and upper width, are expressed with ${l}_{ijk}$, ${m}_{ijk}$, and ${u}_{ijk}$, respectively.
  10 $${l}_{ij}={\left(\prod_{k=1}^{K} {l}_{ijk}\right)}^{1/K}; {m}_{ij}={\left(\prod_{k=1}^{K} {m}_{ijk}\right)}^{1/K}; {u}_{ij}={\left(\prod_{k=1}^{K} {u}_{ijk}\right)}^{1/K}$$
  in which $K$ is the total number of respondents.
4. **Chang’s extent analysis:** This step ensures the consideration of inherent vagueness and uncertainty in experts’ cast of minds. To obtain the fuzzy quantities, crisp mathematical notations are used in this respect. Let $X=\left\{{x}_{1},{x}_{2},{x}_{3},\dots \dots \dots ,{x}_{n}\right\}$ and $U=\left\{{u}_{1},{u}_{2},{u}_{3},\dots \dots \dots {u}_{m}\right\}$ be the object and goal sets, respectively. Extent analysis values represented by $m$ is attained for each object that is considered to implement the extent analysis for each goal, i.e., ${u}_{i}$. More details regarding the steps of extent analysis can be found in Chang (1996).
5. **Sensitivity analysis:** Sensitivity analysis helps to ensure the final solution is not overly sensitive to small changes in the input data. The pertinent literature contains different implementations of sensitivity analysis, such as changing and/or shifting the criteria weights to observe the changes in the attained output (Jain et al. 2018; Dogan 2021), changing the fuzziness degrees to discover the variations in the importance levels of the criteria considered (Ekmekcioğlu et al. 2022b). In the present study, we tested five different fuzziness degrees, i.e., 1.2, 1.4, 1.6, 1.8, and 2.0, along with the base scenario with a degree of 1, to ensure that our decision-making framework is resistant against the fuzziness degree. Hence, a deeper understanding of the problem being solved is provided and the stability and robustness of the proposed scheme was tested.

**Table 2:** Linguistic scales and triangular fuzzy reciprocals of AHP and FAHP

Linguistic variables	AHP		Fuzzy AHP	
	Importance	Value for reciprocals	Triangular fuzzy numbers $({l}_{ij},{m}_{ij},{u}_{ij})$	Triangular fuzzy reciprocals $({1/u}_{ij},{1/m}_{ij},{1/l}_{ij})$
Equally important	1	(1/1)	(1,1,1)	(1,1,1)
Intermediate value	2	(1/2)	(1,2,3)	(1/3,1/2,1)
Moderately important	3	(1/3)	(2,3,4)	(1/4,1/3,1/2)
Intermediate value	4	(1/4)	(3,4,5)	(1/5,1/4,1/3)
Important	5	(1/5)	(4,5,6)	(1/6,1/5,1/4)
Intermediate value	6	(1/6)	(5,6,7)	(1/7,1/6,1/5)
Very important	7	(1/7)	(6,7,8)	(1/8,1/7,1/6)
Intermediate value	8	(1/8)	(7,8,9)	(1/9,1/8,1/7)
Extremely important	9	(1/9)	(9,9,9)	(1/9,1/9,1/9)

**Table 3:** Profile of the experts who attended the pairwise survey

Experts’ ID	Position	Affiliation	Work experience (years)
E1	Professor	University	21
E2	Professor	University	23
E3	Professor	University	24
E4	Infrastructure manager	Water and sewerage administration	13
E5	Geology engineer	Water and sewerage administration	10
E6	Technical office	Water and sewerage administration	25
E7	Technical office	Water and sewerage administration	20
E8	Control engineer	Municipality	15
E9	General manager	Private sector	15
E10	Engineer	Private sector	10

**Table 4:** Randomness Index (Saaty 1977)

*n*	1	2	3	4	5	6	7	8	9	10
Random Index	0	0	0.52	0.89	1.11	1.25	1.35	1.40	1.45	1.49

### Weighted overlay analysis

The weighted overlay analysis (WOA) combining multiple thematic raster layers into a single layer is one of the commonly adopted spatial GIS tools. The WOA takes both the pre-defined criteria weights pertaining to different spatial layers and their actual values into account to evaluate the combined impacts of the thematic layers for producing overall results. Compared to the weighted sum tool that is another knowledge-based empirical raster processing method (Khatun et al. 2022), the WOA possesses a greater degree of as it allows not only facilitating the standardization of input factors through conversion to a user-defined scale (Ahmad et al. 2022) but also enabling the specification of benefit factors and cost factors for which higher values indicate more and less suitability, respectively.

The WOA have extensively been used in a wide range of purposes, such as groundwater potential zoning (Kayal et al. 2022), identifying artificial groundwater recharge zones (Kaliraj et al. 2015; Sandoval and Tiburan 2019; Aju et al. 2021), and groundwater quality mapping (Ben Brahim et al. 2022). The current research utilized the WOA rationale via the WhiteboxTools (Lindsay 2014) for QGIS to conduct the assessments regarding the MAR suitability areas. Hence, the criteria weights acquired through the FAHP analysis and the thematic layers affecting the MAR suitability were combined by considering the constraining layer refined by means of the well-known DRASTIC approach. It is worth mentioning that, before implementing the WOA, all thematic layers are ensured in the same projection and standardized to the identical cell size. The calculation procedure of the WOA can be expressed as follows:

11 $$MSL=\sum_{i=1}^{n}{R}_{i}\times {W}_{i}$$

where *MSL* is the MAR suitability index, while *R*_*i*_ and *W*_*i*_ denote the raster layer and the weight of *i*th criteria, respectively.

## Results and discussions

### Groundwater vulnerability assessment

The implementation of recharge structures in areas prone to pollution could have adverse impact on the success of MAR projects. Hence, the delineation and exclusion of the vulnerable regions within the aquifer are of utmost importance. In this study, the DRASTIC method was used as a constraint tool to generate a groundwater vulnerability map. Previous researches have suggested that modifying the scores and weights of the conventional DRASTIC model can result in a robust correlation with certain hydrochemical parameters, such as nitrate (Kazakis and Voudouris 2015; Barzegar et al. 2019; Balaji et al. 2021). Accordingly, this paper presents a comparison of the vulnerability maps generated through the original DRASTIC and three modified DRASTIC models that are based on three MCDA approaches, namely AHP, FAHP, and SWARA.

Table 5 illustrates the scores and weights employed in the investigated vulnerability models. In terms of ratings, the models seem to exhibit a level of importance consistent with that of the conventional DRASTIC model for the criteria ranges except the SWARA model. The DRASTIC-SWARA model, as proposed by Torkashvand et al. (2021), exhibits differences in the ratings assigned to the classes of the *D*, *R*, and *T* layers compared to those of other models. Furthermore, significant variations in the weights assigned to the DRASTIC parameters can be observed across the different models. To exemplify, based on the DRASTIC-original, *D*, *I*, and *R* are the most significant parameters with weights of 5, 5, and 4, respectively. In the DRASTIC-SWARA, layers *R*, *D*, and *S* represent the main determinant with 0.351, 0.342, and 0.104. Based on the DRASTIC-AHP, *D*, *R*, and *I* complete the podium with 0.236, 0.235, and 0.208, respectively. In reference to the DRASTIC-FAHP model, Table 5 reveals that *I*, *D*, and *S* are the predominant factors with values of 0.223, 0.207, and 0.161, respectively.

**Table 5:** Ratings and weights used for the original DRASTIC, DRASTIC-SWARA, DRASTIC-AHP, and the DRASTIC-FAHP

Layer	Range	DRASTIC-original		DRASTIC-SWARA		DRASTIC-AHP		DRASTIC-FAHP	
	Source:	[1]		[2]		[3], [4], [5], [6]		[7], [8], [9]	
		Rating	Weight	Rating	Weight	Rating	Weight	Total weight	Weight
Depth to water table (m)	1.4–4.5	9	5	0.112	0.342	0.381	0.236	1.862	0.207
	4.5–9.0	7		0.197		0.232		1.448	
	9.0–15.0	5		0.242		0.154		1.034	
	15–22.5	3		0.181		0.097		0.621	
	22.5–30	2		0.130		0.071		0.414	
	> 30	1		0.139		0.063		0.207	
Net Recharge*	9.0–11.0	8	4	0.145	0.351	0.519	0.235	0.712	0.089
	7.0–9.0	5		0.163		0.268		0.445	
	5.0–7.0	3		0.280		0.136		0.267	
	3.0–5.0	1		0.412		0.077		0.089	
Aquifer media	Granular sand	8	3	0.339	0.075	0.525	0.090	1.094	0.137
	Silty gravel	6		0.257		0.211		0.820	
	Silty sand	5		0.202		0.118		0.684	
	Volcanic tuf	4		0.202		0.057		0.547	
	Hisarcık lava	3		0.000		0.063		0.410	
	Pyroclastic rock	2		0.000		0.025		0.273	
Soil media	Sandy	9	2	0.268	0.104	0.276	0.095	1.449	0.161
	Sandy loam	7		0.206		0.218		1.127	
	Sandy clay	6		0.172		0.250		0.966	
	Silty loam	5		0.142		0.150		0.805	
	Silty clay loam	3		0.107		0.054		0.483	
	Clay loam	2		0.105		0.052		0.322	
Topography (%)	0–2	10	1	0.212	0.004	0.418	0.032	0.525	0.053
	2.0–6.0	9		0.194		0.274		0.473	
	6.0–12.0	5		0.193		0.168		0.263	
	12.0–18.0	3		0.193		0.082		0.158	
	> 18	1		0.208		0.058		0.053	
Impact of the vadose zone*	6.0–8.0	8	5	0.545	0.051	0.495	0.208	1.784	0.223
	4.0–6	5		0.455		0.239		1.115	
	3.0–4	3		0.000		0.174		0.669	
	2.0–3	1		0.000		0.092		0.223	
Hydraulic Conductivity (m/day)	43.20–47.52	8	3	0.360	0.071	0.435	0.104	1.055	0.132
	8.64–43.20	6		0.283		0.256		0.791	
	6.48–8.64	4		0.179		0.143		0.527	
	4.32–6.48	3		0.179		0.096		0.396	
	< 4.32	2		0.000		0.070		0.264	

^*^Layers generated by using the Piscopo method; [1] Aller et al. (1987); [2] Torkashvand et al. (2021); [3] Jhariya et al. (2019); [4] Bera et al. (2022); [5] Umar et al. (2022); [6] Karan et al. (2018); [7] Jesiya and Gopinath (2019); [8] Goodarzi et al. (2022); [9]Sresto et al. (2022)

**Fig. 8:**
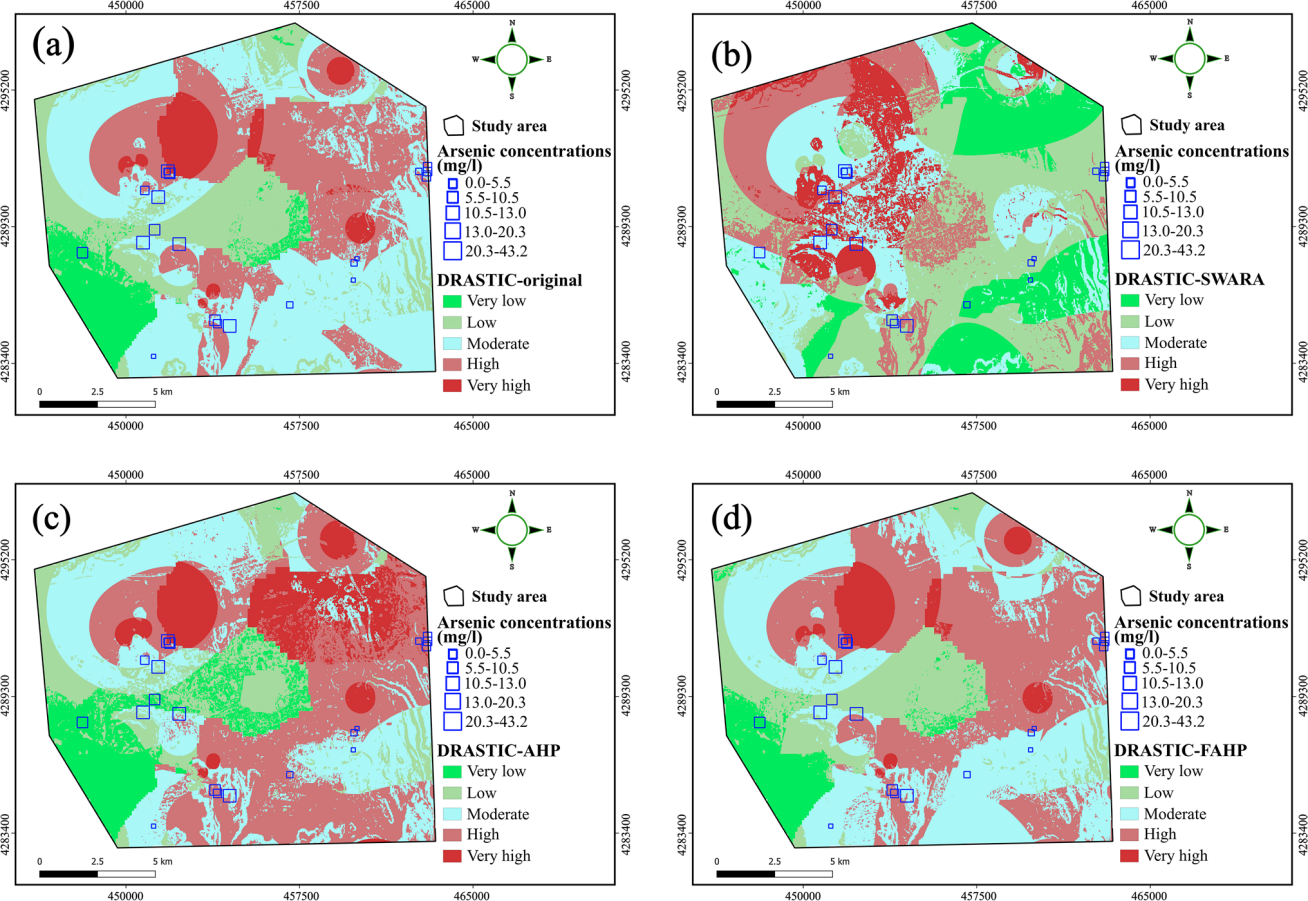
Vulnerability maps derived based on the **a** original DRASTIC, **b** DRASTIC-SWARA, **c** DRASTIC-AHP, and **d** DRASTIC-FAHP

Vulnerability maps were generated based on the various DRASTIC models considered in this study and presented in Fig. 8. The maps were reclassified into five classes: very low, low, moderate, high, and very high. The DRASTIC-original model revealed that a relatively small portion of the study area (5%) was categorized as having a very high vulnerability to groundwater contamination. Furthermore, 28% of the region exhibited high vulnerability, while 41% of the area showed moderate vulnerability. The low and very low vulnerability zones are mainly situated in the central and western parts of the city (Fig. 8a). However, regarding the DRASTIC-SWARA, the low and very low vulnerability areas are more concentrated in the eastern part, with much greater surface coverage for both classes in a way that regions characterized by low and very low vulnerability cover 18% and 30% of the study region, respectively (Fig. 8b). In contrast to the traditional DRASTIC, the high vulnerability zones have shifted to the north western and center parts of the focalized region. It is further worth noting that the DRASTIC-original, AHP, and FAHP models displayed similarities in the obtained vulnerability pattern with only proportions of vulnerability classes varying (Fig. 8a, c, and d). Nevertheless, the DRASTIC-AHP was the one giving the highest vulnerable regions (high to very high classes) among all considered models with around 45% of the study area unsuitable for MAR activities. Conversely, the SWARA vulnerability map has the largest suitable regions (very low to moderate classes) with nearly two-third of the focalized region fit to host MAR structures.

**Fig. 9:**
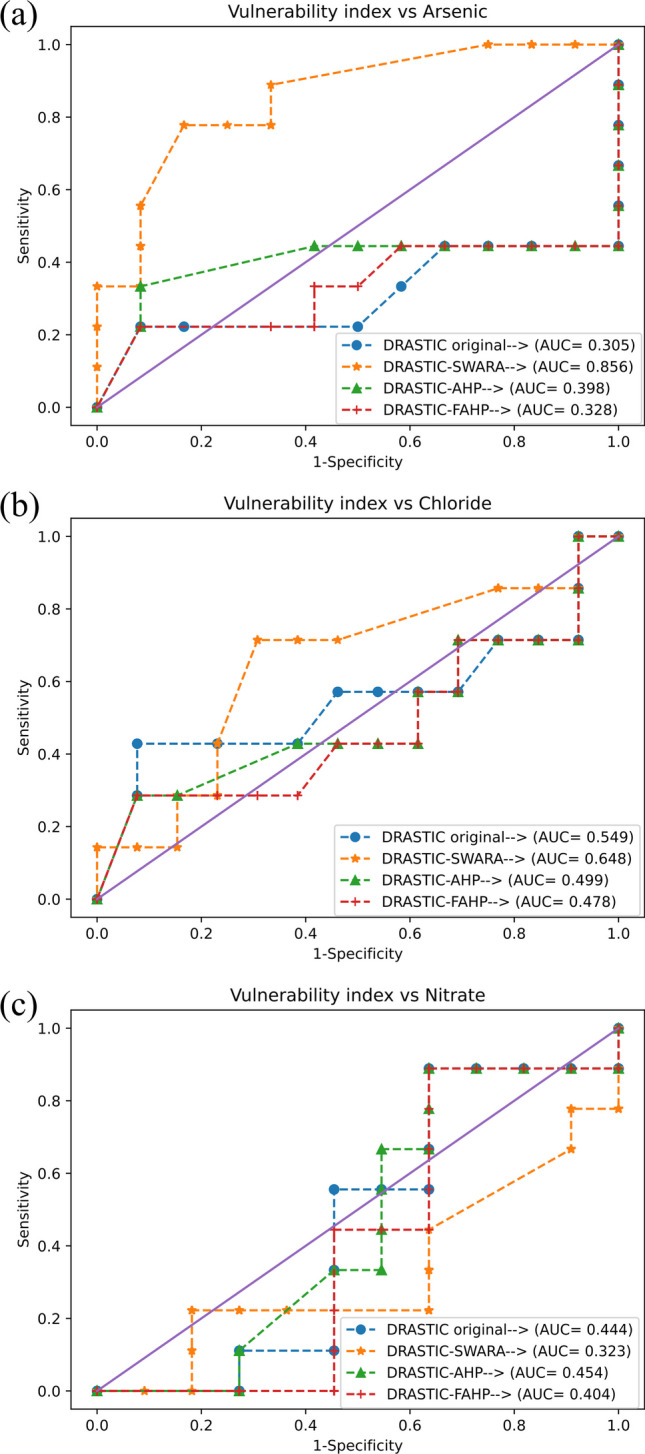
ROC curves and obtained AUC scores of the DRASTIC models based on different hydrochemical indicators (As, Cl^−^, and NO_3_^−^)

Furthermore, validation is an important procedure in groundwater vulnerability mapping as it enables the assessment of the accuracy and reliability of the proposed models (Kumar and Pramod Krishna 2020). In the validation of DRASTIC models, the correlation between the determined vulnerability index and the NO_3_^−^ has long been adopted as a means of verification, assuming that the absence of nitrate characterizes the initial state of the aquifer. However, Hamza et al. (2015) argued that the validations that rely solely on NO_3_^−^ concentration are inadequate and proposed the inclusion of data regarding other hydrochemical parameters in order for ensuring the robustness of the analysis. Consequently, recent studies have considered using multi-pollutants found in the groundwater to validate their models. Karan et al. (2018), for instance, used sulfate (SO_4_^2−^) and iron (Fe) concentrations as validation parameters. Momejian et al. (2019) employed total dissolved solids (TDS) and Cl^−^ to compare the accuracy of two different vulnerability indexes. Hasan et al. (2019) used the concentrations of three hydrochemical parameters, i.e., NO_3_^−^, EC, and chromium (Cr), for validating their models. In a similar vein, Yu et al. (2022) adopted the same number of parameters, including NO_3_^−^, Mg^2+^, and chemical oxygen demand (COD), to validate the accuracy and compare the proposed DRASTIC models. In line with these studies, the present research selected NO_3_^−^, As, and Cl^−^ to compare the output maps generated by the original and modified DRASTIC models and determined the superior approach. The results, shown in Fig. 9, depict the ROC curves and calculated AUC values for the various vulnerability indexes in relation to NO_3_^−^, As, and Cl^−^ concentrations. Although the DRASTIC-AHP outperformed its counterparts with an AUC of 0.454 with regard to the NO_3_^−^ concentration, based on the obtained AUC values (Fig. 9c), any model yielded satisfactory performance with no significant relationship with NO_3_^−^. Referring to the Cl^−^, the DRASTIC-SWARA produced the highest correlation with 0.648 followed by the DRASTIC-original, DRASTIC-AHP, and DRASTIC-FAHP with AUC values of 0.549, 0.499, and 0.478, respectively. Given As, likewise the comparisons made according to the Cl^−^, the DRASTIC-SWARA surpassed other vulnerability models with an AUC of 0.856 (Fig. 9a and b). Overall, based on the AUC classification proposed by Yesilnacar and Kadir (2005), the DRASTIC-SWARA showed superior performance compared to the others, with demonstrating average and very good performance category according to the Cl^−^ and As, respectively. As a result, the DRASTIC-SWARA was selected to generate the constraint map, and therefore, used to spot vulnerable zones in the aquifer.

### Decision criteria importance level

**Fig. 10:**
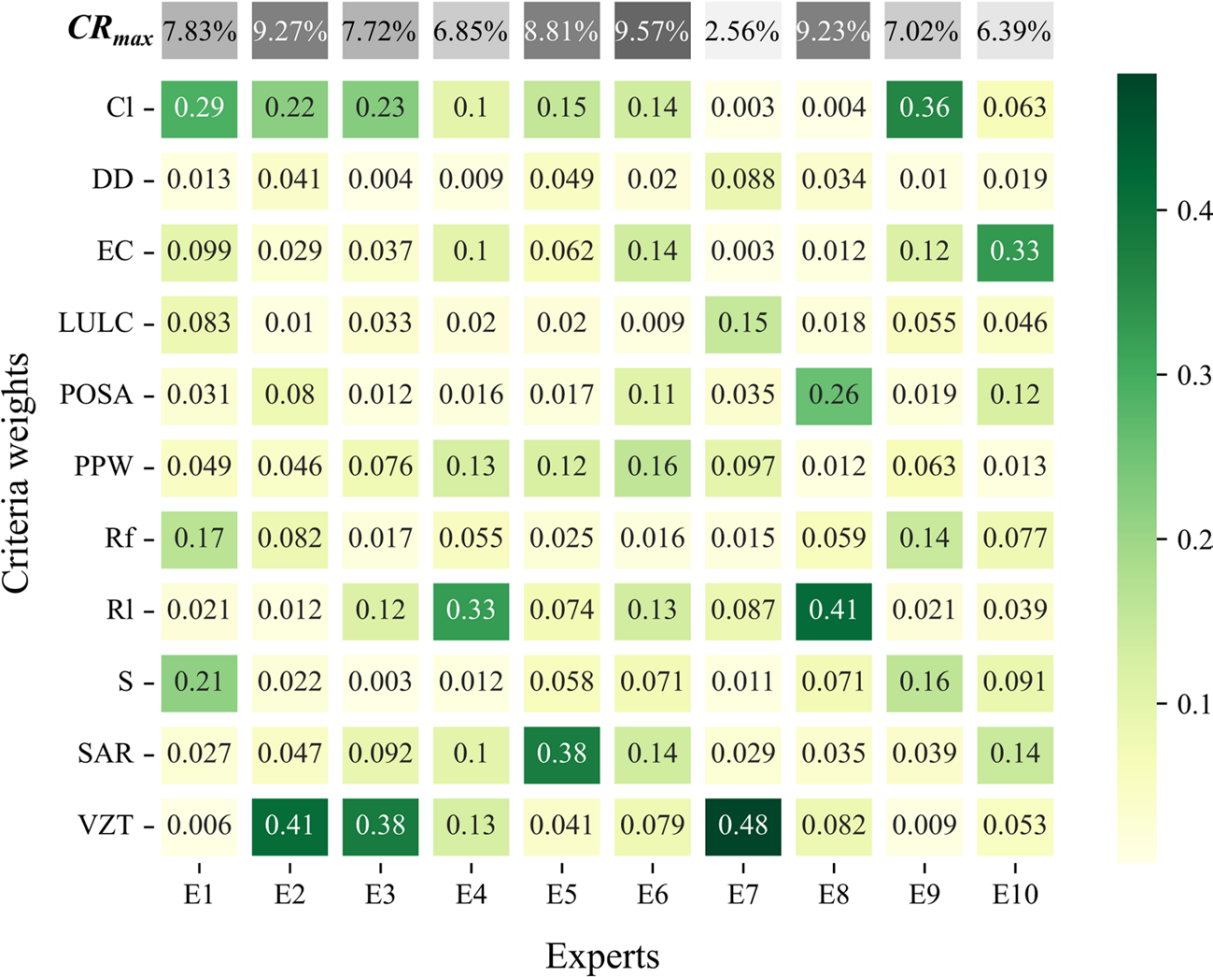
Heat map of the criteria weights and the maximum CR values obtained through different participants

This research identified the relative importance of the clusters and the corresponding sub-criteria based on the FAHP analysis. However, as one of the most significant aspects of the GIS-MCDA approaches is to explore the weights of criteria considered and the fact that the MCDA rationale directly rely on the preferences of the experts who performed the pairwise comparison surveys (Malczewski and Rinner 2015), it is essential to ensure the consistency of the judgments of the survey participants. In this regard, the CR values for each expert and every pairwise comparison made by them were measured to verify the reliability of the analysis. The heat map presented in Fig. 10 illustrates the distributions of the weights as well as the maximum CR value reached by the different participants. As can be seen from the figure that all the experts who attended the pairwise comparison surveys provided consistent preferences since the CR values obtained for each expert were found below the threshold of 10%. Hence, the obtained maximum CR values illustrate the validity and consistency of the conducted pairwise surveys.

In examining the weights determined by various experts, the insights drawn from Fig. 10 shed light on the nuanced perspectives brought forth by the expert questionnaires during the pairwise comparison surveys. It is worth mentioning that the divergence in the backgrounds of the experts have produced substantial variations in the assigned weights to different criteria. For instance, perceivable differences among E1, E2, and E3 all working as a Professor in universities underscore not only the influence but also significance of disciplinary expertise on decision-making. E2 and E3, both affiliated with the Hydrogeology department, accorded significant importance to VZT in groundwater protection, reflected by their respective global weights of 0.41 and 0.38, whereas E1 having Environmental Engineering background allocated a comparatively lesser weight of 0.006 to VZT, prioritizing instead the quality of groundwater (Cl). This disparity elucidates how disciplinary perspectives can shape the prioritization of criteria within the decision-making process. Additionally, the assessment made by E7, another expert whose inclination towards VZT aligns with the hydrogeological engineering paradigm, further emphasizes the disciplinary influence on decision outcomes. Finally, the corresponding facts highlight the need for constructing comprehensive decision-making scheme encompassing participants from diverse disciplines, as it provides delicate interpretation and application of decision-making outcomes in complex domains such as groundwater management.

Table 6 shows the global weights determined by the FAHP analysis for the main clusters and each criterion. It was found that the groundwater quality cluster had the highest weight with 37.4%, followed by the surface and groundwater protection cluster with 25.2% and 19.4%, respectively. On the other hand, the hydrometeorology cluster was deemed to be the least important with a weight of 17.9%. Regarding the surface criteria group, the POSA was identified as the most influential parameter, with a global weight of 9.46%, while R_l_ and VZT ranked first in the hydrometeorology and groundwater protection clusters, respectively. Despite this, Cl^−^ was found as the most significant criterion in the overall assessments, followed by VZT, SAR, R_l_, and POSA, respectively. Also, one notable observation is that despite the groundwater quality having a significantly higher weight than the other three clusters, the EC criterion demonstrated the least contribution (6.15%) to the overall comparisons, succeeded by PPW and DD.

**Table 6:** Weights determined for each decision parameter through the FAHP analysis and the ratings assigned to criteria classes

Clusters	Weight	Criteria	ID	Weight	Classes	Ratings	Global weight
Surface	25.2%	Slope (%)	S	6.61%	0–1	5	0.42
					1–3	4	0.33
					3–5	3	0.25
					5–10	2	0.17
					> 10	1	0.08
		Land use/land cover	LULC	7.13%	Forest and seminatural areas	5	0.28
					Arable land, permanent crops, pastures, and heterogenous agricultural areas	4	0.22
					Urban fabric, green urban areas, sports, and leisure facilities	3	0.17
					Industrial, commercial, and transport units	2	0.11
					Mine, dump, and construction sites	1	0.06
		Drainage density (km/km^2^)	DD	2.04%	0–0.38	5	0.10
					0.38–1.04	4	0.08
					1.04–1.73	3	0.06
					1.73–2.58	2	0.04
					2.58–4.46	1	0.02
		Potential occurrence of shallow groundwater (TWI + NDVI)	POSA	9.46%	Good	5	0.47
					Fair	3	0.28
					Poor	1	0.09
Hydrometeorology	17.9%	Rainfall (mm/year)	R_l_	10.88%	422–397	5	0.54
					367–397	4	0.44
					346–367	3	0.33
					314–346	2	0.22
					272–314	1	0.11
		Runoff (mm)	R_f_	7.04%	> 368	5	0.35
					341–368	4	0.28
					319–341	3	0.21
					290–319	2	0.14
					234–290	1	0.07
Groundwater protection	19.4%	Proximity to public wells (m)	PPW	3.69%	> 800	5	0.18
					500–800	4	0.15
					300–500	3	0.11
					150–300	2	0.07
					0–150	1	0.04
		Vadose zone thickness (m)	VZT	15.73%	> 40	5	0.79
					25–40	4	0.63
					10–25	3	0.47
					5–10	2	0.31
					0–5	1	0.16
Groundwater quality	37.4%	Electrical conductivity (µS/cm)	EC	6.17%	345–1000	5	0.31
					1000–1257	4	0.25
					1257–1590	3	0.19
					1590–2143	2	0.12
					2143–3620	1	0.06
		Sodium adsorption ratio (meq/l)	SAR	14.51%	0.16–1.26	5	0.73
					1.26–1.90	4	0.58
					1.90–2.57	3	0.44
					2.57–4.13	2	0.29
					4.13–7.58	1	0.15
		Chloride (mg/l)	Cl^−^	16.75%	9–64	5	0.84
					64–114	4	0.67
					114–162	3	0.50
					162–350	2	0.34
					> 350	1	0.17

**Fig. 11:**
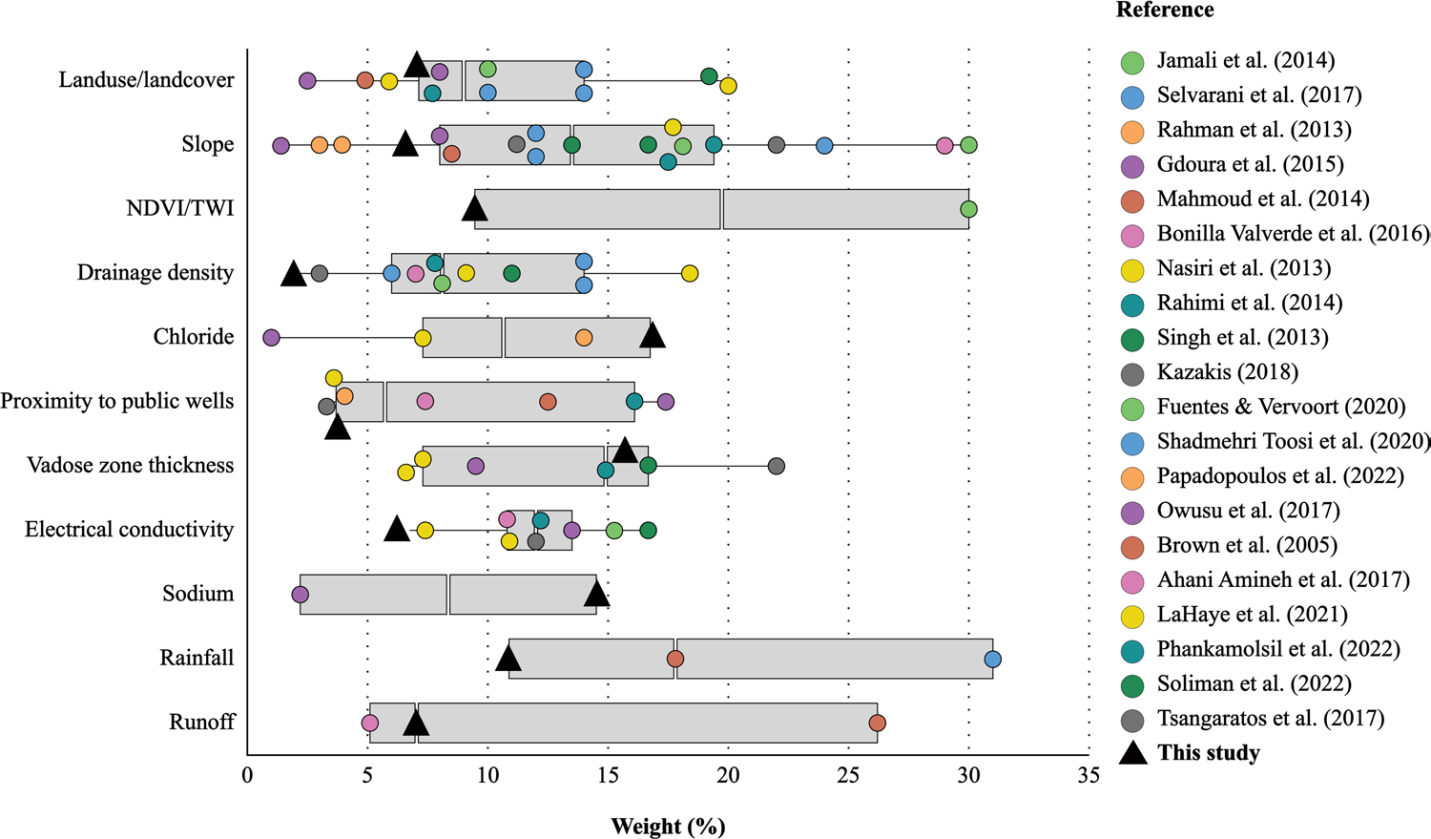
Comparative analysis of the criteria weights obtained in the present study with those attained in similar studies focusing on MAR potential mapping

Furthermore, to bolster the validity of our findings, we conducted a comparison of the weights derived in our study with those obtained in relevant literature on the subject, as illustrated in Fig. 11. It is evident that, for the weights attributed to VZT, LULC, Rf, and PPW, they align closely with or approximate the median of their respective criteria sets (Fig. 11). Despite slope being a commonly utilized criterion in MAR potential mapping (Mouhoumed et al. 2024), the weight assigned to it in our study closely corresponds to the first quartile of the set. Notably, in our investigation, Cl^–^ emerged with the highest level of importance in the decision framework, a result which aligns with the findings of Fuentes and Vervoort (2020) and Rahman et al. (2013), both of whom accorded significant importance to groundwater quality parameters. Conversely, EC received a lower level of significance, which is consistent with existing literature that similarly attributes a lesser importance to EC in delineating suitable MAR implementation sites (Malekmohammadi et al. 2012; Nasiri et al. 2013; Sandoval and Tiburan 2019; Hussaini et al. 2021). Nonetheless, minor disparities were observed between the weights assigned to decision criteria in our study and those documented in existing literature. This variance may be attributed to the size of decision parameters utilized in our study and the diversity of backgrounds among the expert panel engaged in the pairwise survey (Mouhoumed et al. 2023). Given that MCDA methods heavily rely on the subjective judgments of participants in pairwise questionnaires, variations in weights assigned to the same criterion across different studies within the literature are expected. Figure 11 serves as a pertinent illustration of this phenomenon, highlighting a notable interquartile range for some criteria set.

**Fig. 12:**
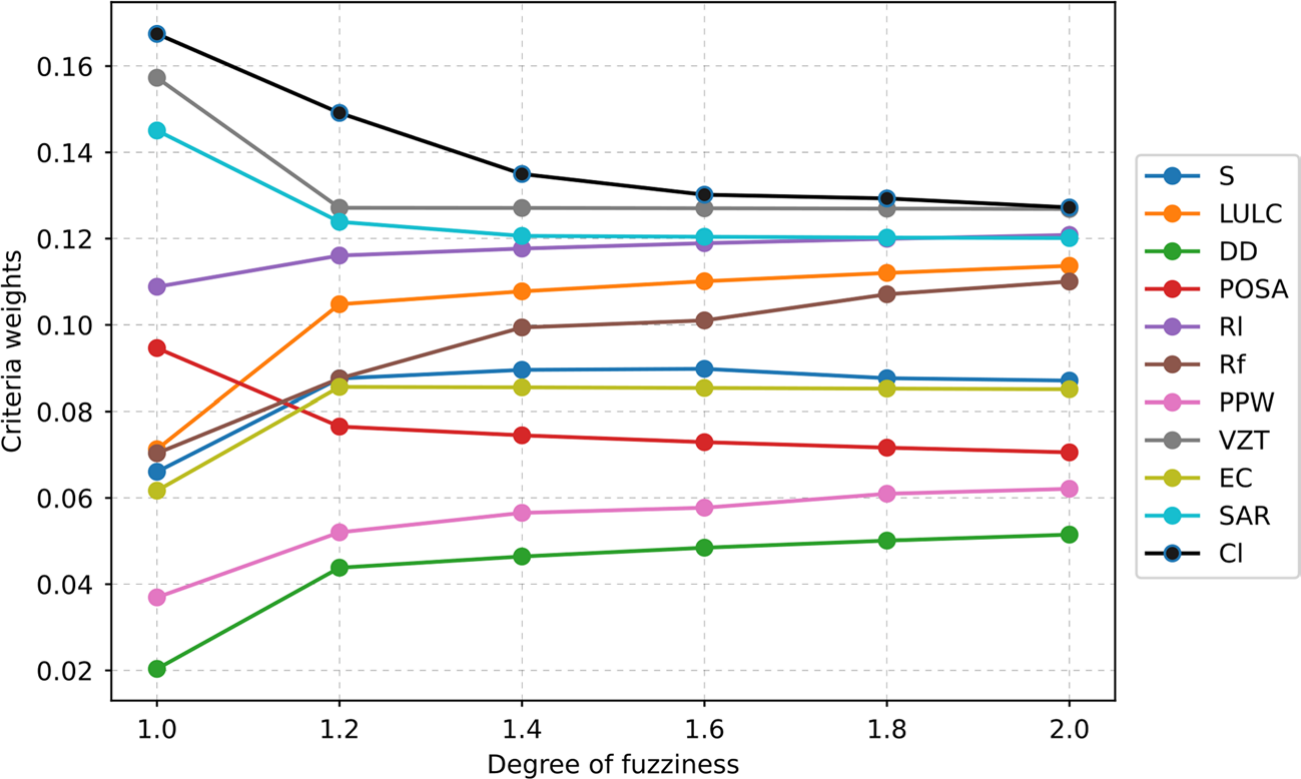
Sensitivity analysis with respect to different fuzziness degrees

MAR potential mapping stands as a widely employed method to evaluate the viability of deploying artificial recharge techniques for addressing aquifer-related issues in specific regions. However, the lack of field measurements or locating successful MAR projects in many countries poses a challenge to model validation in these studies (Sallwey et al. 2019). Although some researchers have attempted to validate their decision frameworks indirectly by examining hydrochemical parameters, water table fluctuations, or numerical models (Russo et al. 2015; Tiwari et al. 2017; Zhang et al. 2019; Fuentes and Vervoort 2020; Arshad et al. 2022), such endeavors are relatively infrequent. Consequently, the majority of scholars suggest the consistency checks and sensitivity analysis as sufficient tools for assessing the robustness of the proposed GIS-MCDA framework for MAR mapping (Rahman et al. 2013; Kazakis 2018). This is because the weighting process, which is often subjective and highly depends on human judgments, constitutes the primary source of uncertainty in such frameworks. Therefore, a rigorous and transparent sensitivity analysis can enhance the credibility and reliability of the MAR suitability mapping results. In line with, the present study examined the sensitivities of the employed criteria to the fuzziness degrees in order to underpin the stability of the FAHP decision framework and its corresponding outcomes. The decisions made by the respondents in such qualitative attempts play a decisive role on the attained outputs. The literature suggests reflecting the potential ambiguities in decision-making mechanism by taking their distinct characteristics (e.g., attitudes) into account (Balusa and Gorai 2019). In this sense, subjectivity in expert judgments can be reconciled and the robustness of the analysis is ensured allowing to accomplish more tailored and effective decision-making mechanism. Hence, different fuzzification factors (or fuzziness degrees) are used to minimize the bias in decision outcomes. Accordingly, along with the implementation of the traditional triangular fuzzy membership functions, the present research further delved into the impact of different degrees of fuzziness illustrating the confidence of the adopted framework (Keprate and Ratnayake 2016). To achieve this, different fuzziness degrees ranging from 1.2 to 2.0 (with a step of 0.2) were considered, and the resulting changes in the criteria weights were explored (Fig. 12). The figure quite revealing that except for the POSA criterion which exhibit slight variation with the change in the degree of fuzziness, there were no major changes in the rankings of decision criteria. Hence, the outcomes of the efforts devoted to sensitivity analysis suggested that the proposed FAHP approach posed significant stability.

### Artificial recharge site suitability assessment

The integration of thematic layers to generate the overall suitability map was carried out in the prioritization process. In the present study, the 11 raster layers were standardized to a common scale and used as input variables for the WOA algorithm. The existing literature recognized the utility of several standardization techniques, e.g., typical fuzzy membership, AHP method, linear and step functions, and rating technique, which ensure the decision layers are in a similar scale (Rahman et al. 2012; Kamangar et al. 2019; Fuentes and Vervoort 2020; Papadopoulos et al. 2022). Due to its practical utilization (Kazakis 2018; Fuentes and Vervoort 2020; Shadmehri Toosi et al. 2020), this study adopted the rating technique to simplify the analysis. A rating factor of 5 was assigned to cells having a higher potential for dry well success, while those posing least suitability were represented by 1 (Table 6). Moreover, the WOA in the WhiteboxTools enables the inclusion of a restriction layer during the process, making it an ideal and useful tool for the development of suitability maps that automatically incorporates constraint layers.

**Fig. 13:**
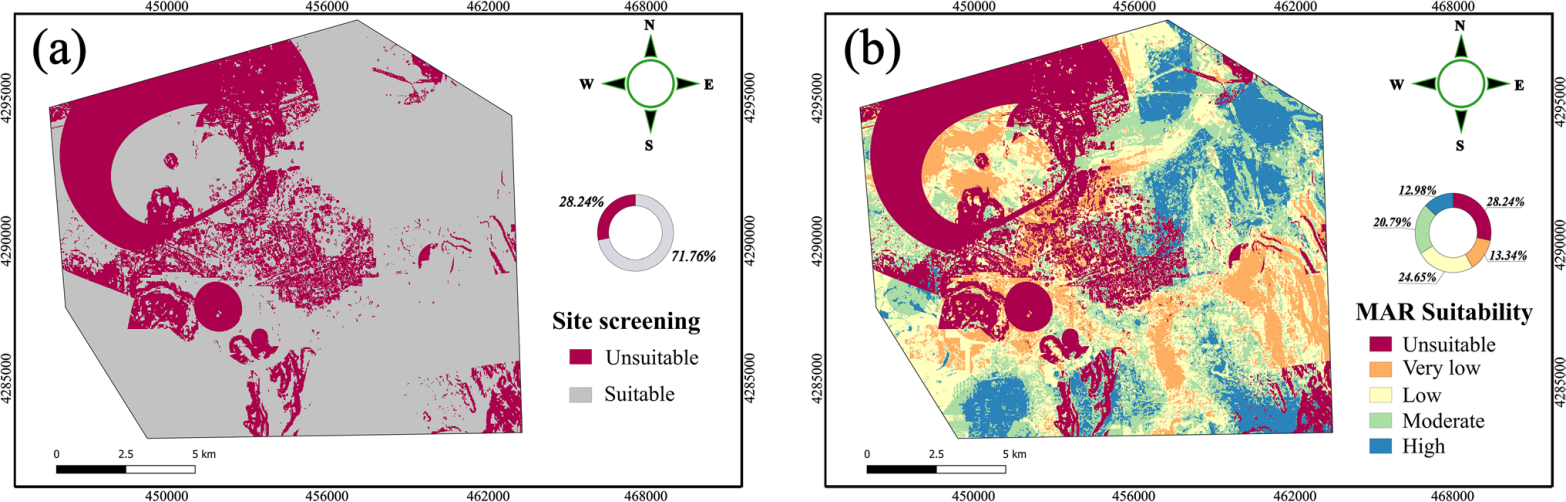
Generated maps through **a** the constraint and **b** final drywell suitability process

Utilizing the DRASTIC-SWARA method, vulnerable regions of the aquifer were evaluated, and areas classified as high and very high vulnerability levels were deemed unsuitable for MAR structures. In contrast, areas categorized as moderate, low, and very low vulnerability were considered suitable for the recharge activities. The overall outcome of this process is illustrated in Fig. 13a, which reveals that 28.24% of the study area is unsuitable for MAR infrastructure, and the remaining 71.76% is suitable. According to Fig. 13a, the majority of the unsuitable areas are located in the northwestern and central regions of Kayseri city. To further refine the suitability analysis, areas identified as suitable for recharge operations were taken into account to calculate the MSL index through the WOA. The obtained MSL index was classified into four distinct categories, namely very low, low, moderate, and high. The resulting final suitability map is presented in Fig. 13b. The findings showed that 13.34% and 24.65% of the investigated area are classified as very low and low suitability, respectively. The reason for such low suitability in those identified regions could be linked to the presence of low groundwater quality with high Cl^−^, SAR, and EC concentrations especially in the center and northwestern part of the aquifer. Additionally, those areas are characterized by the presence of shallow water table (less than 10 m) implying a thin vadose layer and thereby low contaminant attenuation potential in those zones. A substantial 20.79% of the study area was classified as having moderate suitability, concentrated primarily in the northeastern and southern parts of the city. The regions classified as having high suitability, which constitute 12.98% of the total area, are predominantly located also in the northeastern and southern parts of the city. These areas are characterized by low to very low Cl^−^ and SAR concentrations, thick vadose zones, and good to fair POSA, all of which enhance their suitability potential, making them favorable sites for constructing drywells. Furthermore, the absence of the least weighted LULC classes such as industrial, commercial, and urban fabric as wells as the presence of flat terrain further assists the suitability potential of those zones for MAR application.

### Limitations and future attempts

Although the developed suitability map offers insightful contributions regarding the MAR potential of the region, the developed map alone does not guarantee the success of any drywell projects in the promising zones but rather needs to be used in the pre-design stage or as a base map. In a similar vein, since drywells offers moderate attenuation potential, stormwater runoff quality could be regarded as another important parameter. The types and concentrations of pollutants in the vicinity of the focalized regions where recharge facilities are potentially be installed need to be thoroughly investigated prior to the full-scale drywell design projects. Furthermore, subsurface heterogeneity is another crucial parameter as it controls the infiltration capacity of the vadose zone as well as the fate and transport of contaminants. To ensure the successful implementation of drywell designs, investigating the heterogeneity of the vadose zone is essential. Therefore, along with generating the final suitability map, it is imperative to conduct on-site infiltration tests and numerical modeling to validate the corresponding assessments.

Despite important contributions made by this study, it still contains some limitations in terms of both conceptual and methodological aspects. From the conceptual facet, additional relevant parameters in drywell sitting could be considered in the follow-up attempts. In regions having rich data, more low-resolution surface (e.g., soil texture) as well as aquifer (e.g., transmissivity) parameters could be included among the decision criteria. Moreover, the uneven spatial distribution of wells in the study area could lead to biased interpolations (Li and Heap 2011). Specifically, the IDW method, employed as an interpolation technique to derive certain criteria within the decision framework, operates under the assumption that nearby points exert a greater influence on interpolated values than distant points. Thus, addressing the uneven distribution of wells in future attempts is imperative to minimize uncertainty in the interpolation process. Furthermore, it is pertinent to acknowledge that this study relied on data from a decade ago to generate some layers of the framework (e.g., soil permeability, hydraulic conductivity, arsenic, nitrate, SAR). Given the potential temporal variations in these parameters, it is essential to re-operate the decision framework using more recent data in subsequent attempts. Socio-economic factors such housing and population densities and urban development could also be added in future drywell site delineation models. Additionally, although drywell usage is common in urbanized areas, their application sector expanded in recent years and they started to be used in agricultural land in conjunction with other MAR technologies. Therefore, by refining the list of criteria, the proposed methodology could be given a shoot in agricultural MAR (agMAR) suitability mapping.

Concerning the methodological point of view, while the present research adopted the FAHP rationale to explore the criteria importance, further attempts can inspect the utilization of other MCDA techniques (such as analytical network process). Likewise, we incorporated the triangular fuzzy membership functions into the FAHP analysis, whereas future studies may discover other fuzzy membership functions, such as gaussian, trapezoidal, and sigmoid functions. In addition, the comparison of other prioritization techniques, e.g., technique for order of preference by the similarity-to-ideal-solution (TOPSIS), VIseKriterijumsa Optimizacija I Kompromisno Resenje (VIKOR), and PROMETHEE, could be a fertile for follow-up attempts. Along with efforts devoted to identify the criteria weights and alternative prioritization, interested scholars may direct their attempts towards illustrating the perception differences of the stakeholders involving in the decision-making processes.

## Concluding remarks

The success of any MAR project is contingent upon the selection of appropriate locations for the recharge structures, especially in urban aquifers where anthropogenic activities endanger the groundwater systems making in terms of pollution. Likewise, despite multifarious benefits of the MAR structures, locating them areas less susceptible to groundwater pollution inhibits maximizing the recharge benefit. Therefore, this study proposed a comprehensive framework that combines groundwater vulnerability and MAR site suitability assessments to identify areas with the greatest potential for the installation of MAR technologies. To perform the groundwater vulnerability assessment of the study area, different DRASTIC methods were investigated and compared, and the best performing option was used to generate the vulnerability layer and constraint map. In addition, being relevant to the drywell designs, a total of 11 decision criteria which were extracted from an extensive literature survey and confirmed to the experts were selected at first, and then, they were standardized and weighted by means of the pairwise comparison survey before being used as input factors for the WOA algorithm.

The results of the vulnerability assessment indicated that the DRASTIC-SWARA had the best match with As and Cl^−^ based on the AUC values of 0.856 and 0.648, respectively. Furthermore, the outcomes of the site screening process demonstrated that 28.24% of the investigated area was unsuitable for recharge activities due to considerable groundwater vulnerability. The FAHP procedures followed to perform criteria weighting showed that the groundwater quality parameters (i.e., particularly Cl^−^ and SAR) were significant in identifying areas suitable for MAR sitting. On the other hand, the proximity to public wells and drainage density were deemed as the least influential criteria regarding the MAR implementation. Sensitivity analysis further revealed that the proposed FAHP model was stable and robust, as there were no significant changes in the rankings of decision parameters with varying degrees of fuzziness. Based on the final suitability assessment, the northeastern and southern parts of the study area have shown high to moderate suitability to implement the MAR structures. Therefore, incorporating drywells into the existing infrastructures, particularly the stormwater drainage network of the city, in the corresponding areas would be a viable option to direct the runoff into the subsurface and replenish the stressed unconfined aquifer of the region.

Still, this study pinpointed the integration of vulnerability assessment into the suitability mapping to exclude vulnerable areas of the aquifer and prioritize the remaining regions with regard to the MAR suitability. The proposed approach presents a valuable basis for municipal authorities and decision-makers to evaluate large regions and concentrate on limited promising areas for further investigations, while mitigating the risk of MAR failure associated with vulnerable zones. This methodology can be adopted by researchers and practitioners globally in other urban aquifers under stress to develop appropriate MAR mapping strategies. In doing so, the decision criteria can further be tailored according to specific technology used to address the acknowledged problem. By thoroughly investigating the subsurface heterogeneity and verifying the final suitability maps with different approaches, the proposed decision-making mechanism offers a reliable basis for the planning and implementation of MAR structures.

## Data Availability

Data will be made available on request.
